# Effect of Short-Term Supplementation with Ready-to-Use Therapeutic Food or Micronutrients for Children after Illness for Prevention of Malnutrition: A Randomised Controlled Trial in Nigeria

**DOI:** 10.1371/journal.pmed.1001952

**Published:** 2016-02-09

**Authors:** Saskia van der Kam, Nuria Salse-Ubach, Stephanie Roll, Todd Swarthout, Sayaka Gayton-Toyoshima, Nma Mohammed Jiya, Akiko Matsumoto, Leslie Shanks

**Affiliations:** 1 Médecins Sans Frontières, Amsterdam, Netherlands; 2 Ecole de Santé Publique, Centre de Recherche en Politiques et Systèmes de Santé-Santé Internationale, Université Libre de Bruxelles, Brussels, Belgium; 3 Médecins Sans Frontières, Barcelona, Spain; 4 Institute for Social Medicine, Epidemiology and Health Economics, Charité- Universitätsmedizin, Berlin, Germany; 5 Department of Paediatrics, Usmanu Danfodiyo University Teaching Hospital, Sokoto, Nigeria; Makerere University Medical School, UGANDA

## Abstract

**Background:**

Globally, Médecins Sans Frontières (MSF) treats more than 300,000 severely malnourished children annually. Malnutrition is not only caused by lack of food and poor infant and child feeding practices but also by illnesses. Breaking the vicious cycle of illness and malnutrition by providing ill children with nutritional supplementation is a potentially powerful strategy for preventing malnutrition that has not been adequately investigated. Therefore, MSF investigated whether incidence of malnutrition among ill children <5 y old could be reduced by providing a fortified food product or micronutrients during their 2-wk convalescence period. Two trials, one in Nigeria and one in Uganda, were conducted; here we report on the trial that took place in Goronyo, a rural region of northwest Nigeria with high morbidity and malnutrition rates.

**Methods and Findings:**

We investigated the effect of supplementation with ready-to-use therapeutic food (RUTF) and a micronutrient powder (MNP) on the incidence of malnutrition in ill children presenting at an outpatient clinic in Goronyo during February to September 2012. A three-armed, partially-blinded, randomised controlled trial was conducted in children diagnosed as having malaria, diarrhoea, or lower respiratory tract infection. Children aged 6 to 59 mo were randomised to one of three arms: one sachet/d of RUTF; two sachets/d of micronutrients or no supplement (control) for 14 d for each illness over 6 mo. The primary outcome was the incidence of first negative nutritional outcome (NNO) during the 6 mo follow-up. NNO was a study-specific measure used to indicate occurrence of malnutrition; it was defined as low weight-for-height z-score (<−2 for non-malnourished and <−3 for moderately malnourished children), mid-upper arm circumference <115 mm, or oedema, whichever came first.

Of the 2,213 randomised participants, 50.0% were female and the mean age was 20.2 (standard deviation 11.2) months; 160 (7.2%) were lost to follow-up, 54 (2.4%) were admitted to hospital, and 29 (1.3%) died. The incidence rates of NNO for the RUTF, MNP, and control groups were 0.522 (95% confidence interval (95% CI), 0.442–0.617), 0.495 (0.415–0.589), and 0.566 (0.479–0.668) first events/y, respectively. The incidence rate ratio was 0.92 (95% CI, 0.74–1.15; *p* = 0.471) for RUTF versus control; 0.87 (0.70–1.10; *p* = 0.242) for MNP versus control and 1.06 (0.84–1.33, *p* = 0.642) for RUTF versus MNP. A subgroup analysis showed no interaction nor confounding, nor a different effectiveness of supplementation, among children who were moderately malnourished compared with non-malnourished at enrollment. The average number of study illnesses for the RUTF, MNP, and control groups were 4.2 (95% CI, 4.0–4.3), 3.4 (3.2–3.6), and 3.6 (3.4–3.7). The proportion of children who died in the RUTF, MNP, and control groups were 0.8% (95% CI, 0.3–1.8), 1.8% (1.0–3.3), and 1.4% (0.7–2.8).

**Conclusions:**

A 2-wk supplementation with RUTF or MNP to ill children as part of routine primary medical care did not reduce the incidence of malnutrition. The lack of effect in Goronyo may be due to a high frequency of morbidity, which probably further affects a child’s nutritional status and children’s ability to escape from the illness–malnutrition cycle. The duration of the supplementation may have been too short or the doses of the supplements may have been too low to mitigate the effects of high morbidity and pre-existing malnutrition. An integrated approach combining prevention and treatment of diseases and treatment of moderate malnutrition, rather than prevention of malnutrition by nutritional supplementation alone, might be more effective in reducing the incidence of acute malnutrition in ill children.

**Trial Registration:**

clinicaltrials.gov NCT01154803

## Abstract 2

A trial in Nigeria reveals no reduction of malnutrition in children who are treated with ready-to-use food following a bout of acute illness. Compared to reductions seen in a similar trial in Uganda, the children in this setting were more malnourished initially.

## Editors' Summary

**Background:**

Malnutrition among children is a global public health problem. Malnourished children have about a 10-fold greater risk of death than well-nourished children and, worldwide, more than 70 million children have moderate or severe acute malnutrition. Acute malnutrition causes wasting—a wasted child has a low weight for his or her height compared to the World Health Organization Child Growth Standards, which chart the growth of a reference population. Multiple factors can cause malnutrition among children, including not having enough to eat and being given the wrong types of food. In addition, recurrent infections are a major cause of malnutrition among children in many tropical countries. Common infections such as malaria, diarrhea, and lower respiratory tract infections all negatively affect the growth of children. Moreover, inadequate nutrition limits recovery from infection and the ability of the immune system to fight off infection, thereby setting up a vicious cycle of malnutrition and illness.

**Why Was This Study Done?:**

One way to interrupt this cycle and reduce the global burden of malnutrition among children might be to ensure that ill children receive a nutritional supplement such as a ready-to-use therapeutic food (RUTF) or a micronutrient powder (MNP) at the same time as their prescribed medical treatment. RUTF, which is based on peanut butter, contains dried skim milk, vitamins, and micronutrients and is supplied as a paste that is eaten directly. Micronutrients are vitamins and minerals that are needed in small quantities for immune system function and for good health. MNP is consumed by mixing it with porridge or other meals. In this randomized controlled trial undertaken by Médicins San Frontières (MSF, a not-for-profit organization that delivers emergency medical aid worldwide), the researchers investigate whether short-term provision of RUTF or MNP prevents the development of malnutrition among ill children under 5 y old living in Goronyo, a rural region of northwest Nigeria where up to 15% of children are acutely malnourished and where levels of illness among children are high.

**What Did the Researchers Do and Find?:**

The researchers randomly assigned 2,213 non-malnourished and moderately malnourished children who visited outpatient clinics in Goronyo with malaria, diarrhea or lower respiratory tract infection to be given RUTF or MNP by their caregivers for 14 d following each illness over a 6-mo period or to receive no supplement. The primary trial outcome was the incidence of the first negative nutritional outcome (NNO) during follow-up (the proportion of the population experiencing NNO during follow-up). NNO was defined as a weight-for-height z-score below −2 or −3 for non-malnourished and moderately malnourished children, respectively (this score compares a child’s weight-for-height with that of a reference population; a z-score of −2 or less indicates acute malnutrition), a mid-upper arm circumference of less than 115 mm, or nutritional oedema (swelling caused by malnutrition). The incidence rates of NNO were 0.522, 0.495, and 0.566 first events/y in the RUTF, MNP, and control groups, respectively. The incidence rate ratio for RUTF versus control was 0.92, a nonsignificant reduction in the incidence of malnutrition (a nonsignificant change in an outcome could have occurred by chance). Provision of MNP also did not significantly reduce the incidence of malnutrition.

**What Do These Findings Mean?:**

These findings show that, among non-malnourished and moderately malnourished children living in Goronyo, Nigeria, provision of RUTF or MNP as part of routine primary medical care during convalescence following malaria, diarrhea, or a lower respiratory tract infection did not reduce the incidence of malnutrition. Because RUTF is popular with caregivers and children, the lack of blinding in this trial (participants knew whether they were being given RUTF, MNP or no supplement) may limit the accuracy of these findings. Moreover, these findings only apply to ill children and cannot be extrapolated to healthy children. Notably, a companion trial undertaken by MSF in Kaabong, Uganda found that short-term supplementation with RUTF reduced the incidence of malnutrition following illness. The researchers suggest that the lack of effect of nutritional supplementation in Goronyo may be because the duration and/or dose of supplementation was insufficient to mitigate the effects of high levels of illness and pre-existing malnutrition present in this setting. Thus, they suggest, an integrated approach that combines the prevention and treatment of diseases with the treatment of moderate malnutrition might be necessary to break the illness–malnutrition cycle among children living in Goronyo and similar settings.

**Additional Information:**

This list of resources contains links that can be accessed when viewing the PDF on a device or via the online version of the article at http://dx.doi.org/10.1371/journal.pmed.1001952.

- A *PLOS Medicine* Research Article by van der Kam et al. describes the companion trial investigating the effect of short-term food supplementation for children in Uganda after illness on the incidence of malnutrition
- More information about this trial is available
- The MSF website contains information about malnutrition around the world; "Starved for Attention" is an international multimedia campaign launched in 2010 by MSF and the VII Photo agency to rewrite the story of childhood malnutrition
- The not-for-profit organization UNICEF, which protects the rights of children and young people around the world, provides detailed information on nutrition among children and statistics on malnutrition among children; a short 2013 article describes UNICEF efforts to reduce malnutrition in Nigeria
- The WHO Child Growth Standards are available (in several languages)
- The World Food Programme is the world’s largest humanitarian agency fighting hunger worldwide
- The Emergency Nutrition Network (ENN) is an interactive website for knowledge sharing and peer support to strengthen the evidence and know-how for effective nutrition interventions in countries prone to crisis and high levels of malnutrition
- The International Lipid-based Nutrient Supplements (iLiNS) project aims to help prevent malnutrition by developing Lipid-based Nutrient Supplements and test their efficiency and by collecting and sharing publications on LNS.

## Introduction

The global burden of malnutrition among children is staggering; with an estimated 8% prevalence (51.5 million children) of moderate acute malnutrition (MAM) and 2.9% prevalence (18.7 million children) of severe acute malnutrition (SAM) [1]. Malnourished children have a higher risk of mortality, ranging from a 3-fold increased risk for the moderately malnourished to a nearly 10-fold increase for the severely malnourished [2]. However, the cause of malnutrition in most tropical countries is multi-factorial, involving not only inadequate nutrition and caring practices, but also recurrent infections. It has long been known that malnourished children have a higher risk on severe outcome of morbidity and that ill children have a higher risk on malnutrition. In 1968, almost half a century ago, the World Health Organization (WHO) suggested a synergistic relation between malnutrition and infection, but not much was known about its mechanisms [3].

In the following decades, insights developed that infections aggravate malnutrition by decreasing appetite, inducing catabolism, and increasing both the demand for nutrients as well as nutrient losses. When increased needs are not compensated by increased consumption, weight loss during an infection is frequent [4,5]. Failure to return to normal nutritional status after an illness increases a child’s susceptibility to further infections, perpetuating a cycle towards further reduced nutritional state [6–11].

It has been shown that diarrhea and malaria are associated with substantial weight loss and that supplementation could promote recovery between infections [6,12].

From the nineties onwards, it was increasingly recognized that protein–energy malnutrition has a depressing effect on the immune system and that even mild degrees of malnutrition begin to adversely affect immunity, which consequently increases morbidity and mortality [5,13].

Research advanced on the mechanisms of the immune system in general, and of the role of individual micronutrient deficiencies—such as zinc and vitamin A—in decreased immunity in particular. Further research focused on how supplementation with these individual micronutrients could prevent illness, reduce the severity of illness, or decrease morbidity and mortality [14–17].

As many micronutrients play a role in the immune system, growth and recovery, a deficiency in a few nutrients or even in one could be the limiting factor. Therefore, nutritional fortification with multiple micronutrients were tested, but the impact on diarrhea and other morbidity were mixed [18,19]. In addition, multi-micronutrients have some effect on linear growth, but the effect was small [19–21]. Reports on the effects of lipid-based nutrient supplements (LNS) on morbidity and weight gain also gave mixed results [22–24]. However, there are indications that LNS supplementation decreases mortality [25].

The question whether supplementation with micronutrients or multi-micronutrients, with or without food, is effective in preventing malnutrition and further morbidity in ill children is unanswered, as the results of research are mixed and foremost lacking. Above all, current studies targeted the general population of children, not specifically ill children.

Until now, it is the general expectation that good nutrition during convalescence of an illness improves recovery. Therefore, WHO recommends that caretakers give their children daily additional nutritious food for 14 days after the onset of illness [26–28].

In resource-poor settings—the typical context of the Médecins Sans Frontières (MSF) programs—this recommendation is likely to be ineffective, as caregivers often lack the healthy ingredients and other resources to implement it. A more effective strategy to reduce disease-related malnutrition in resource-poor areas may be to provide ill children with nutritional supplements at the point of care alongside medical treatment.

MSF treated 292,221 severely malnourished children and 71,471 moderately malnourished children in 2012. It is imperative that MSF explores appropriate ways to prevent malnutrition, of which an effective strategy might be providing nutritional supplementation alongside medical treatment to ill children.

MSF piloted the potential of supplementation to ill children in Democratic Republic of Congo (DRC). This research showed that children with malaria gained weight faster during the period of supplementation when given 14 d of ready-to-use therapeutic food (RUTF), although this difference was not seen at 4 wk follow-up [29].

Because of these promising results, MSF investigated whether supplementation of a nutritional supplement—either a lipid-based fortified food or micronutrients—to children with diarrhea, malaria, and/or lower respiratory tract infection (LRTI) during their 2-wk convalescence period is effective in reducing the incidence of malnutrition over a period of 6 mo. Two trials, one in Nigeria and one in Uganda, were conducted. Here we report on the trial that took place in Nigeria; the trial in Uganda is published elsewhere [30].

## Methods

### Approval

The trial was registered at clinicaltrials.gov number NCT01154803. The full protocol and the statistical analysis plan (SAP) can be accessed in the supporting information files (S1 Text and S2 Text). The study received approval, collaboration and cooperation of Nigerian health authorities (Sokoto State Director Primary Health care at 24 August 2010 and Goronyo LGA and the Sokoto State Research Ethical Committee at 24 September 2010). Approval was also obtained from the MSF Ethics Review Board (23 December 2010).

### Setting

The study was implemented in the town of Goronyo, Sokoto State, in the northwest of Nigeria. Because of a high frequency of severe malnutrition among children and its contribution to high morbidity, this site was chosen to investigate whether nutritional supplementation to ill children would significantly decrease the incidence of malnutrition.

Goronyo is rural and primarily dependent on agriculture (including irrigated) and animal husbandry for its livelihood, with income also derived from trade and small-scale manufacturing. A variety of crops are grown, including millet, sorghum, rice, maize, groundnuts, cowpeas, okra, onions, spinach, and tomatoes [31]. The climate has one rainy season from May to October, which is also the traditional hunger season and malaria season, while from January to June it can be very hot.

A qualitative study in the Goronyo Local Government Area in 2009 concluded that malnutrition is linked to inappropriate infant and young child feeding practices; substandard levels of or access to health services, water supply, hygiene, and sanitation; inadequate (health) education; and a poor understanding of the importance of food quality, quantity, and diversity [32]. A high proportion of children under 5 y were not consuming protein-rich and nutrient-dense foods on a daily basis; for example, only 35% of diets included milk and dairy products, and only 8% of diets included fish and poultry [33].

Surveys conducted in March 2009 and March 2010 (during the hot, dry season) showed a global acute malnutrition (GAM) prevalence of 14.8% and 11.5%, respectively, and a SAM prevalence of 4.9% and 2.6%, respectively [33,34]. A study by Save the Children Fund concluded that a chronic existence of malnutrition is linked to a marginal economic situation and under-resourced health care system combined with gender inequality and poor weaning practices in northern Nigeria [35].

The 2009 survey found that 51% of the children reported having an illness (including fever, diarrhoea, and cough) at some time during the previous 14 d, suggesting a high burden of disease that likely exacerbates an already precarious problem of malnutrition. The prevalence of malnutrition among those children who reported an illness in the previous 14 days was 23%, compared with 7% among nonsick children (*p* < 0.001), illustrating the relation between malnutrition and disease [34].

Médecins Sans Frontières-Operational Centre Amsterdam (MSF-OCA) has provided medical support to Sokoto State Hospital Goronyo since 2008. The activities included outpatient clinics and hospital-based medical care for children, including a therapeutic feeding programme with intensive inpatient and four outpatient facilities. In 2012, MSF treated 9,505 children for severe malnutrition in Goronyo. MSF withdrew from Goronyo in February 2013 because of security issues.

### Study Objectives and Endpoints

The aim of this three-armed, partially-blinded, randomised controlled trial was to determine the effectiveness of 14 days of nutritional supplementation with a RUTF or a micronutrient powder (MNP) given to children 6–59 mo of age and diagnosed with and treated for malaria, diarrhoea, and/or LRTI in reducing the incidence of acute malnutrition compared with a control group during a follow-up of 6 mo. Given the complexity of malnutrition in the trial’s study population, a study-specific primary endpoint event was compiled. This is called negative nutritional outcome (NNO) and, depending on nutritional status on enrolment, was defined as moderate and/or severe acute malnutrition (MAM and/or SAM). It was defined as: weight-for-height z-score <−2, MUAC <115 mm, or nutritional oedema, whichever occurred first for non-malnourished children; and weight-for-height z-score <−3, MUAC <115 mm, nutritional oedema (SAM), or weight loss >10% from baseline for moderately malnourished children (weight-for-height z-score <−2). Secondary outcomes included changes in anthropometric indicators, morbidity, and mortality.

### Study Population and Randomisation

Children aged 6 to 59 mo diagnosed with one or more of the three study diseases (malaria, diarrhoea, LRTI) were included in the study when living within approximately 60 min walking distance from the clinic and intending to remain in the area for the duration of the 6-mo follow-up. Children who met the criteria for SAM (defined as weight-for-height z-score <−3, MUAC <115 mm, or nutritional oedema), were exclusively breastfed, had a severe disease (for example, severe malaria, severe pneumonia, or severe anaemia), had a sibling enrolled in the study, or were offspring of staff of the study were excluded.

Children were randomised in a 1:1:1 ratio to one of three intervention groups. During the 6-mo follow-up, children in the RUTF and MNP groups received nutritional supplements for 14 d whenever they were diagnosed with at least one of the three study diseases, with a maximum of 14 d of supplementation in any 28-d period. The MNP contained only micronutrients, according to the United Nations (UN) formulation [36]. The MNP MixMe (DSM Ltd, Switzerland) was mainly used, but owing to a production problem MixMe was replaced by the “vitamin and mineral powder” (Piramal Healthcare Ltd, India) for several follow-up visits from 1 October 2012 to the end of the study.

Children in the MNP group received two doses per d to ensure a micronutrient composition comparable to one sachet of RUTF (Plumpynut, Nutriset, France) (Table 1). Caretakers were instructed to mix MNP in the meal (for example, porridge) just before consumption. All groups (including the control group) received health education, including the message that following an illness, a child should eat one extra healthy meal per day for 2 wk.

**Table 1:** Nutritional supplements’ composition per serving.

Nutrients	1 sachet RUTF^1^	2 sachets MNP^2^
Energy (kcal)	500 kcal	
Protein (g; % total energy)	11.6 (10%)	
Lipid (g; % total energy)	29.5 (56%)	
Calcium (mg)	276	
Phosphorus (mg)	276	
Potassium (mg)	1,022	
Magnesium (mg)	84.6	
Zinc (mg)	12.9	8.2
Copper (mg)	1.6	1.1
Iron (mg)	10.6	20.0
Iodine (μg)	96	180
Selenium (μg)	27.6	34.0
Sodium (μg)	<267	
Vitamin A (μg)	840	800
Vitamin D (μg)	15	10
Vitamin E (μg)	18.4	10.0
Vitamin C (mg)	49	60
Thiamine (mg)	0.55	1.0
Riboflavin (mg)	1.66	1.0
Niacin (mg)	4.88	12.0
Pyridoxine (mg)	0.55	1.0
Cobalamin (μg)	1.7	1.8
Folic Acid (μg)	193	300
Vitamin K (μg)	19.3	
Biotin (μg)	60	
Pantothenic Acid (mg)	2.85	

MNP: micronutrient powder; RUTF: ready-to-use therapeutic food.

^1^Sachet contains 92 g.

^2^2 sachets contain 2 g.

The sample size was based on the assumption of a baseline/control group incidence rate of first NNO event of 0.20 within 6 mo. A 30% reduction, considered a clinically and operationally relevant improvement, would result in a targeted incidence rate of 0.14 in 6 mo in each of the two treatment groups. Using a Poisson regression model, 80% power at a 0.05 significance level, and an assumed dropout rate of approximately 10%, a sample size of 734 children was needed in each group (that is, 2,202 in total for all three groups).

Simple randomisation was based on a computer-generated randomisation list made by an expert independent of the study. Participants entering the study received a study number, and only the staff member giving the supplement was able to connect the study number to the treatment group. All other study staff (including clinicians diagnosing illnesses and deciding on giving an allocation) were blinded to allocation. The statistician was unblinded after completion of the analysis.

### Procedures

Patients were screened in the regular outpatient clinic on eligibility. Potential participants were referred to the adjacent study clinic for further inclusion procedures.

Activities at inclusion included provision of information and obtaining written consent of caretakers and their husbands. In case the caretaker was illiterate the consent form was read out loud and consent was given by thumbprint in the presence of a witness. The enrolled participants were followed after the first 14 d and then monthly during 6 mo (24 wk). If a child was ill, they were invited to come whenever they felt that was needed. At every visit, the health and nutritional status of the participant was assessed.

Anthropometric measurements—weight, height, oedema, and MUAC—were taken at every visit to the study clinic. Two types of electronic weighing scales were used to measure weight: SECA model 354, with a precision of 10–20 g, and SECA 869, a mother/baby scale with a precision of 100 g. Length or height (change of measuring position at 85 cm of height) was measured using a precision height board (infant-child-adult measuring board ICAM, aluminium, precision 1 mm, Promes). MUAC was measured using a standard MUAC tape (MSF, precision 2 mm).

Malaria was diagnosed by conducting a malaria rapid diagnostic test (Paracheck). All newly enrolled participants were tested on enrollment; during the study, participants would get a malaria test upon indication (fever or history of fever), except during a malaria epidemic in 2012 when all participants were tested at every visit from 1 June onwards.

Diarrhoea was defined as three or more loose stools (bloody or nonbloody) in 24 h and was diagnosed by mothers’ report. LRTI was diagnosed by the following algorithm: children with cough or having difficulty breathing, an increased respiratory rate (for children aged 12–59 mo >50 breaths/min and for 6–11 mo >40 breaths/min), and absence of chest in-drawings were diagnosed as nonsevere LRTI; chest in-drawing was considered as a sign of severe LRTI, needing referral to the hospital.

All children received standard care and treatment according to current national protocols. Uncomplicated malaria was treated for 3 d with artemether–lumefantrine (Lumartem, Cipla, India). Acute watery diarrhoea was treated using oral rehydration therapy with low osmolarity oral rehydration salts and zinc according to WHO guidelines, regardless of the supplement received. LRTI was treated with amoxicillin 80–100 mg/kg/d for 5 d. The first treatment was given under medical supervision, and the use of and need for the drug were explained to the mother.

Participants presenting with other nonsevere diseases were treated in the study clinic according to the national protocol. Participants with measles received 2 wk of RUTF, as this is considered standard of care for measles cases; these children were kept in the study, and normal rules were applied for allocation for a follow-up supplement.

At every visit, health education was provided. This included advice on prevention and treatment of malaria and diarrhoea and the advice to give an ill child an extra nutritious meal for 14 d.

Home visitors supported the study by reminding the caretakers to come to the appointments, urging follow-up absentees to return, and reporting on deaths that occurred at home. When a participant was found to have died, the caretaker was invited to the study clinic for an interview. Otherwise, the family would be visited by a nurse for an interview. Any death was immediately reported and reviewed by a national paediatrician, principal investigator, and medical director of MSF.

Compliance was measured by questionnaires, asking how many times and for how many days the participant consumed the supplement. In addition, the caretaker was asked to return the full or empty sachets of each distribution. Finally, after the study, three focus groups discussions were held with caretakers who had completed the trial to discuss the supplements and medication used, compliance, and any barriers regarding using the supplements.

### Data Analysis

The primary outcome (rates of first NNO) among the treatment groups was analysed by a Poisson regression model including the three intervention groups with contrasts for each two-group comparison. As there were three interventional groups to be compared, a hierarchical test procedure was used to account for multiplicity. If a significant result (significance level 0.05 two-sided) was observed in the RUTF group compared with control, MNP was then compared with control; if this was also statistically significant at 0.05 two-sided, RUTF was subsequently compared with MNP in a noninferiority approach with a noninferiority margin of 2% (that is, allowing a slightly worse result in the MNP than in the RUTF group, statistically significant at 0.025 one-sided). If no significant result was observed in the first or second step, all other comparisons were considered exploratory.

The incidence rate of malnutrition (NNO) is expressed in first event per 365 observation d (events/y) and is extrapolated from the study period of 168 d (24 wk).

Secondary outcomes were analysed using logistic regression or analysis of covariance with treatment group and baseline values as covariates. In addition, adjustment for nutritional status at baseline (moderate malnourished or non-malnourished on enrollment) was applied. A priori defined subgroups were analysed by including interaction terms in the main model.

Any participant attending the first and the last visit was counted as having completed the study. A participant developing severe malnutrition was referred to the therapeutic feeding programme and withdrawn from the study; all data up to the moment of withdrawal was used. When the participant was erroneously included or consent was withdrawn, the participant was withdrawn and the data was not used. When a patient was admitted to the hospital, the hospital visit was monitored and (if necessary) supplementation was temporarily suspended, but the patient was not withdrawn from the study. The primary outcome was also analysed for a per protocol population that excluded participants if they had abandoned the study, had a low compliance, or had measles (patients with measles were provided with RUTF for 2 wk regardless the supplementation arm)

## Results

### Participant Flow

**Fig 1:**
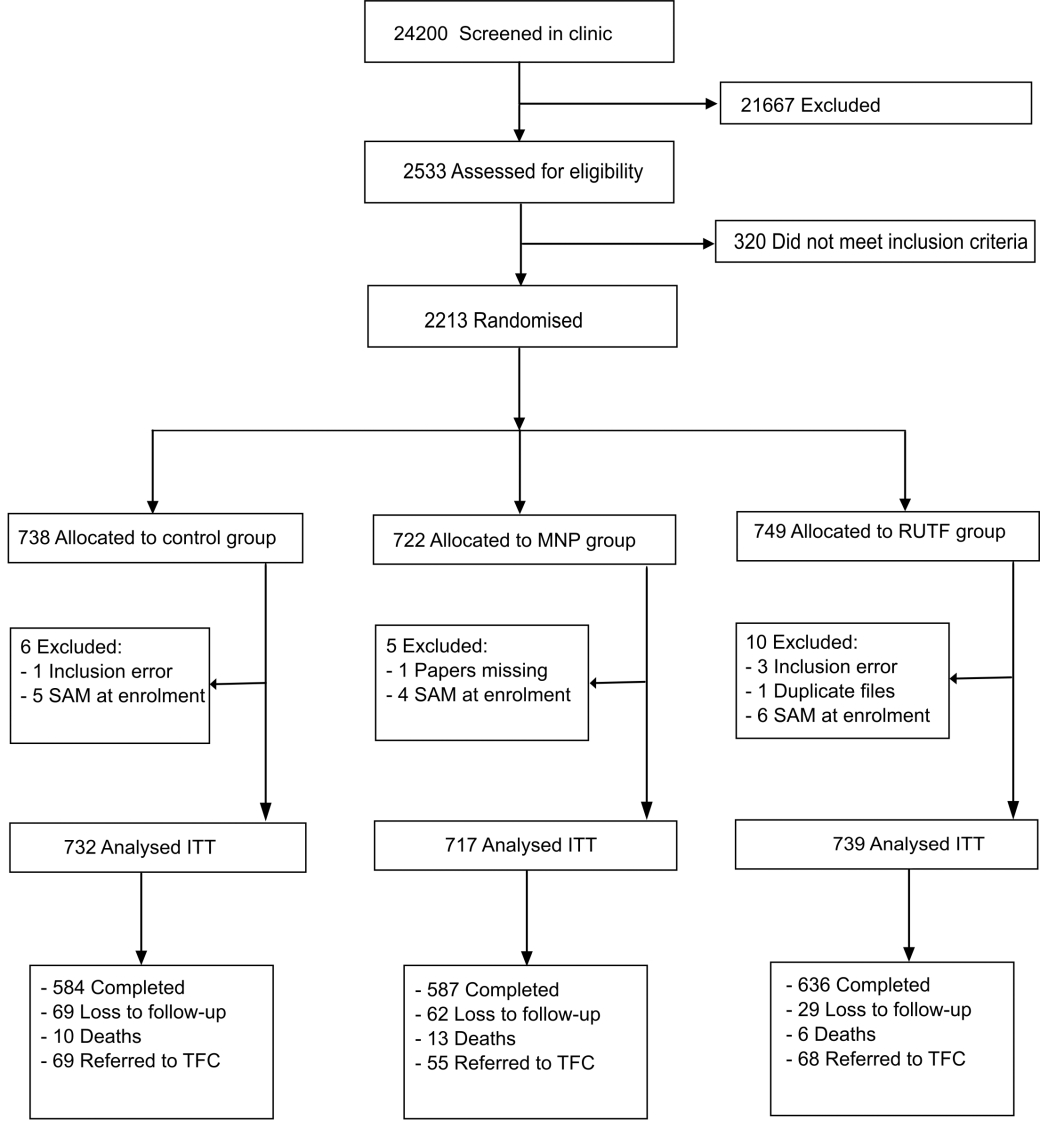
Flow diagram of participants of the supplementation study in Goronyo. MNP, micronutrient powder; RUTF, ready-to-use therapeutic food; SAM, severe acute malnutrition; TFC, therapeutic feeding centre.

Recruitment took place between February 2012 and February 2013. The last patient completed follow-up on 28 February 2013. 24,200 patients younger than 5 y visiting the outpatient clinic were screened on age, illness, and living area; of these, 5.4% were excluded because of severe malnutrition. 2,533 patients were assessed thoroughly for eligibility. A total of 2,213 children aged between 6 and 59 mo were included, with 25 (1.1%) participants excluded from analysis because of inclusion error (21) and withdrawal of consent (4). 160 (7.2%) participants were lost to follow-up (abandoned the study) (Fig 1). The mean number of d in the study was for RUTF, MNP and control groups was 152.3, 148.1, and 145.4 d, respectively.

### Baseline Characteristics

The average household size was 10.9 people, with 29.4% children younger than 5 y. Most caretakers (2,126; 97.2%) were the mothers of the child, and 187 (8.6%) had primary school or more (completed and not completed) 1725 (78.8%) had attended only an Islamic school. More than 40% of the (male) heads of household made a living through business or retail, while 50% of the female caretakers were business owners. Caretakers also frequently (38.5%) reported being a housewife; this means that the caretakers stay at home, but they might be involved in domestic production of garments or processing foods for sale. A quarter (25%) of the heads of household made a living in agriculture or livestock, and 23.5% had a regular paid job. Three-quarters (76.2%) of the households owned livestock, 21.1% owned cows, over half (60.0%) of the households owned a motorcycle, and 24% owned a generator. Most families (79.3%) had consumed three or more meals on the day before the interview (Table 2).

**Table 2:** Baseline characteristics.

Characteristic		RUTF (*n* = 739)	MNP (*n* = 717)	Control (*n* = 732)	Total (*n* = 2,188)
**Baseline household and caretakers**					
Age (years, mean ± SD)		24.8 ± 6.4	25.1 ± 6.9	24.7 ± 6.2	24.9 ± 6.5
Number in house (mean ± SD)	Children <5 y	3.1 ± 2.0	3.4 ± 2.7	3.2 ± 2.3	3.2 ± 2.4
	Children 5–17 y	2.8 ± 2.9	2.9 ± 3.4	3.0 ± 3.0	2.9 ± 3.1
	Adults ≥18 y	4.8 ± 4.0	5.0 ± 4.2	4.6 ± 3.6	4.8 ± 3.9
Education caretaker (*n* [%])	Islamic school	577 (78.1%)	567 (79.1%)	581 (79.4%)	1,725 (78.8%)
	Primary school ((in-)complete)	42 (5.7%)	30 (4.2%)	35 (4.7%)	107 (4.9%)
	More than primary school	28 (3.8%)	28 (3.9%)	24 (3.3%)	80 (3.7%)
	No formal school/other	92 (12.4%)	92 (12.9%)	92 (12.6%)	276 (12.6%)
Occupation of head of household (*n* [%])	Agriculture/livestock	178 (24.1%)	178 (24.8%)	190 (26.0%)	546 (25.0%)
	Business/shop owner	312 (42.2%)	295 (41.1%)	303 (41.1%)	910 (41.6%)
	Regular paid work	179 (24.2%)	182 (25.4%)	154 (21.0%)	515 (23.5%)
	Daily/seasonal labour	33 (4.5%)	32 (4.5%)	43 (5.9%)	108 (4.9%)
	Housewife/other/unknown	37(5.0%)	30 (4.2%)	42 (5.7%)	109 (5.0%)
Occupation of caretaker (*n* [%])	Agriculture/livestock	14 (1.9%)	26 (3.6%)	17 (2.3%)	57 (2.6%)
	Business/shop owner	368 (49.8%)	357 (49.8%)	368 (50.3%)	1,093 (50.0%)
	Regular paid work	29 (3.9%)	21 (2.9%)	28 (3.8%)	78 (3.6%)
	Daily/seasonal labour	44 (6.0%)	31 (39.3%)	37 (5.1%)	112 (5.1%)
	No occupation/housewife/other	284 (38.4%)	282 (38.2%)	282 (38.5%)	848 (38.8%)
Owns livestock (*n* [%])		562 (76.0%)	536 (74.8%)	569 (77.7%)	1,667 (76.2%)
Owns cows (*n* [%])		161 (21.8%)	158 (22.0%)	143 (19.5%)	462 (21.1%)
Has at least one item (*n* [%])	Watch	568 (76.9%)	555 (77.4%)	549 (75.0%)	1,672 (76.4%)
	Motorbike	454 (61.4%)	444 (61.9%)	415 (56.7%)	1,313 (60.0%)
	Radio	582 (78.8%)	564 (78.7%)	564 (77.0%)	1,710 (78.2%)
	Generator	174 (23.5%)	179 (25.0%)	173 (23.6%)	526 (24.0%)
Number of meals per day (*n* [%])	0–2	165 (22.3%)	104 (19.5%)	148 (20.2%)	453 (20.7%)
	≥3	574 (77.7%)	577 (80.5%)	584 (79.8%)	1,735 (79.3%)
**Baseline children**					
Male sex (*n* [%])		366 (49.5%)	342 (47.7%)	385 (52.6%)	1,093 (50.0%)
Age (months, mean ± SD)		19.7 ± 11.1	20.7 ± 11.2	20.3 ± 11.2	20.2 ± 11.2
Aged <36 mo		637 (86.2%)	614 (85.6%)	624 (85.2%)	1,875 (85.7%)
Breastfeeding at enrolment (n [%])	None	334 (45.2%)	344 (48.0%)	328 (44.8%)	1,006 (46.0%)
	Partial	405 (54.8%)	373 (52.0%)	404 (55.2%)	1,182 (54.0%)
MUAC (mm, mean ± SD)		137.6 ± 10.1	138.8 ± 11.1	137.9 ± 10.8	138.1 ± 10.7
Weight (kg, mean ± SD)		8.5 ± 2.0	8.8 ± 2.0	8.7 ± 2.1	8.7 ± 2.0
Height (cm, mean ± SD)		75.8 ± 8.8	76.7 ± 8.8	76.3 ± 8.8	76.2 ± 8.8
Weight-for-age (z-score, mean ± SD)		−2.0 ± 1.0	−2.0 ± 1.0	−2.0 ± 1.0	−2.0 ± 1.0
Height-for-age (z-score, mean ± SD)		−2.0 ± 1.2	−2.0 ± 1.3	−2.0 ± 1.2	−2.0 ± 1.2
Weight-for-height (z-score, mean ± SD)		−1.3 ± 0.9	−1.3 ± 1.0	−1.3 ± 1.0	−1.3 ± 1.0
Study disease malaria^1^ (*n* [%])		179 (24.2%)	217 (30.3%)	197 (26.9%)	593 (27.1%)
Study disease LRTI^1^ (*n* [%])		148 (20.0%)	159 (22.2%)	158 (21.6%)	465 (21.3%)
Study disease diarrhoea^1^ ^,^ ^2^ (*n* [%])		588 (79.6%)	538 (75.0%)	581 (79.4%)	1,707 (78.0%)
Days ill prior to visit (days, mean ± SD)		4.4 ± 2.9	4.7 ± 3.8	4.5 ± 3.1	4.5 ± 3.3
Recruitment period	Feb–May 2012	378 (51.2%)	351 (49.0%)	337 (46.0%)	1,066 (48.7%)
	June–Sept 2012	361 (48.8%)	366 (51.0%)	395 (54.0%)	1,122 (51.3%)

LRTI: lower respiratory tract infection; MNP: micronutrient powder; MUAC: mid-upper arm circumference; RUTF: ready-to use therapeutic food.

^**1**^No hierarchy in study diseases.

^2^Nonbloody or bloody diarrhoea.

Although children up to 5 y of age were included in the study, most (85.7%) were younger than 3 y. About half (54%) of the participants were partially breastfed. Most (78.0%) of the participants had diarrhoea on enrollment, 27.1% had malaria, and 21.3%.had LRTI (more than one disease could be reported).

Caretakers waited an average of 4.5 d after the onset of illness before seeking help at the outpatient clinic; a quarter of the mothers went to the pharmacy to seek a treatment before their clinic visit. Half of the participants (48.7%) were enrolled in the period February–May 2012, participating mostly during the dry and hot season; 51.3% were enrolled in the period June–September 2012, participating mostly during the cold/rainy season.

There was no clear relevant difference between the supplementation groups as regards baseline characteristics (Table 2).

### Incidence of Malnutrition

The incidence of first NNO event in the RUTF, MNP, and control groups was 0.522, 0.495, and 0.566 events per y, respectively, during the 6-mo follow-up. There was no significant difference between study groups. The incidence rate ratio between RUTF and control was 0.922 (*p* = 0.471), between RUTF and MNP 1.055 (*p* = 0.642) and between MNP and control 0.874 (*p* = 0.242), resulting in a reduction of 0.044 events/y (7.8%), −0.027 events/y (−5.3%) and 0.071 events/y (12.6%) respectively. Analysis of the per protocol (PP) dataset (*n* = 1,748) was not different from the intention to treat (ITT) data set: 0.547, 0.517, and 0.585 events/y, respectively, for the RUTF, MNP, and control groups.

The outcome was stratified according to nutritional status on enrolment (non-malnourished and moderately malnourished). The NNO for children who were non-malnourished on enrollment comprises newly diagnosed moderate and severe malnutrition. The non-malnourished group had an incidence of NNO very close to that for the entire study group (for RUTF, MNP, and control groups: 0.522, 0.480, and 0.581 first events/y, respectively). The incidence of first NNO for children who were moderately malnourished on enrolment (comprising only severe malnutrition) was for RUTF, MNP, and control groups: 0.695, 0.705, and 0.711 first events/y, respectively. There was no significant reduction in the incidence of NNO in the RUTF or MNP group (*p*-value for interaction: 0.771) for non-malnourished or moderately malnourished children (Table 3).

**Table 3:** Incidence of first NNO (negative nutritional outcome) per y.

Incidence of first NNO	*n*	Incidence rate (events/y) (95% CI)			Incidence rate ratio (95% CI)			*p*-Value		
		RUTF	MNP	Control	RUTF versus MNP	RUTF versus control	MNP versus control	RUTF versus MNP	RUTF versus control	MNP versus control
**ITT population**										
All^1^	2,156	0.522 (0.442;0.617)	0.495 (0.415;0.589)	0.566 (0.479;0.668)	1.055 (0.841;1.325)	0.922 (0.741;1.149)	0.874 (0.698;1.095)	0.642	0.471	0.242
Non-malnourished at enrollment^2^	1,608	0.522 (0.434;0.629)	0.480 (0.394;0.586)	0.581 (0.483;0.698)	1.087 (0.829;1.427)	0.900 (0.693;1.168)	0.827 (0.631;1.085)	0.546	0.427	0.170
Moderately malnourished at enrollment^2^ ^,^ ^3^	548	0.695 (0.521;0.928)	0.705 (0.526;0.944)	0.711 (0.535;0.947)	0.986 (0.654;1.488)	0.977 (0.651;1.467)	0.991 (0.658;1.491)	0.947	0.912	0.965
**PP population**										
All^7^	1,748	0.547 (0.460;0.651)	0.517 (0.425;0.629)	0.585 (0.492;0.696)	1.058 (0.829;1.351)	0.935 (0.744;1.174)	0.883 (0.692;1.127)	0.651	0.562	0.319

ITT: intention to treat; MNP: micronutrient powder; NNO: negative nutritional outcome; PP: per protocol; RUTF, ready-to-use therapeutic food.

^1^Adjusted for nutritional status at baseline.

^2^ *p*-Value for interaction = 0.771.

^3^Moderately malnourished: weight-for-height z-score between <−2 and −3 AND mid-upper arm circumference >115 mm.

The NNO for children who were non-malnourished on enrollment comprises newly diagnosed moderate and severe malnutrition. The incidence rate of moderate malnutrition among non-malnourished children was 0.456, 0.439, and 0.550 first events/y for RUTF, MNP, and control groups, respectively; the differences were not significant. The incidence of severe malnutrition among non-malnourished children was 0.130, 0.080, and 0.148 first events/y for RUTF, MNP, and control groups, respectively (MNP versus control: *p* = 0.036) (Table 4).

**Table 4:** Incidence of first moderate and severe malnutrition event among non-malnourished at enrolment per y.

Nutrition status at endpoint	Incidence rate (events/year) (95% CI)			Incidence rate ratio (95% CI)			*p* value		
	RUTF	MNP	Control	RUTF versus MNP	RUTF versus control	MNP versus control	RUTF versus MNP	RUTF versus control	MNP versus control
Moderate^1^	0.456 (0.376;0.556)	0.439 (0.356;0.541)	0.550 (0.456;0.665)	1.041 (0.781;1.387)	0.831 (0.632;1.092)	0.798 (0.603;1.057)	0.785	0.184	0.116
Severe^2^	0.130 (0.091;0.185)	0.080 (0.050;0.148)	0.148 (0.106;0.209)	1.613 (0.875;2.882)	0.873 (0.534;1.425)	0.541 (0.305;0.961)	0.107	0.586	0.036

MNP: micronutrient powder; RUTF: ready-to-use therapeutic food. n = 1608

^1^Moderately malnourished: weight-for-height z-score between −2 and −3 and MUAC >115 mm.

^2^Severe malnutrition: weight-for-height z-score <−3 OR MUAC <115 mm or oedema.

In addition, one-quarter of the participants who were moderately malnourished upon enrollment improved to non-malnourishment without showing any difference between the supplementation groups (RUTF, MNP, and control groups: 25.4%, 25.0%, and 27.3%).

Nutritional oedema is included in the definition of NNO and the definition of SAM. A total of 18 children developed oedema (7, 4, and 7 participants in the RUTF, MNP, and control groups, respectively). In addition, all participants who were moderately malnourished at enrollment and lost 10% of their weight during the study were also diagnosed with severe malnutrition and therefore these were analysed having SAM as outcome.

Subgroup analyses (prespecified) shows that the effect of supplementation on the incidence of NNO was not modified by socioeconomic characteristics, season of enrollment, age of the participant, breastfeeding status, or study disease at enrollment (Table 5).

**Table 5:** Incidence rate of first NNO per y by subgroups, adjusted for nutritional status at baseline.

Subgroup	Value	*n*	*p-Value for (interaction)* ^1^	Incidence rate (95% CI)			Incidence rate ratio (95% CI)		
				RUTF	MNP	Control	RUTF versus MNP	RUTF versus control	MNP versus control
**Caretaker/household**									
Owns livestock	Yes	1,645	0.879	0.529 (0.437;0.639)	0.493 (0.403;0.603)	0.556 (0.460;0.671)	1.073 (0.829;1.391)	0.952 (0.742;1.221)	0.887 (0.685;1.147)
	No	511		0.500 (0.353;0.708)	0.501 (0.353;0.711)	0.608 (0.427;0.866)	0.999 (0.623;1.600)	0.822 (0.516;1.310)	0.823 (0.514;1.317)
Owns cows	Yes	455	0.144	0.470 (0.326;0.479)	0.676 (0.483;0.945)	0.658 (0.459;0.942)	0.696 (0.434;1.116)	0.715 (0.438;1.167)	1.027 (0.646;1.633)
	No	1,701		0.536 (0.445;0.646)	0.449 (0.366;0.551)	0.544 (0.451;0.657)	1.195 (0.921;1.548)	0.985 (0.770;1.259)	0.824 (0.636;1.069)
Education	Low	1,969	0.525	0.530 (0.446;0.630)	0.483(0.402;0.581)	0.583 (0.491;0.692)	1.098 (0.866;1.391)	0.909 (0.724;1.140)	0.828 (0.654;1.047)
	Middle	108		0.454 (0.214;0.964)	0.685 (0.340;1.378)	0.432 (0.193;0.966)	0.663 (0.255;1.721)	1.052 (0.381;2.901)	1.586 (0.589;4.270)
	High	79		0.431 (0.162;1.149)	0.537 (0.223;1.289)	0.310 (0.078;1.240)	0.803 (0.216;2.994)	1.389 (0.254;7.586)	1.730 (0.336;8.915)
Number of meals per day	0–2	448	0.223	0.599 (0.427;0.840)	0.607 (0.417;0.883)	0.482 (0.322;0.720)	0.986 (0.614;1.585)	1.244 (0.759;2.038)	1.260 (0.752;2.112)
	≥3	1,708		0.501 (0.414;0.607)	0.469 (0.385;0.572)	0.587 (0.489;0.705)	1.068 (0.825;1.383)	0.854 (0.667;1.092)	0.799 (0.621;1.028)
**Child**									
Age	<36 mo	1,848	0.481	0.570 (0.479;0.679)	0.520 (0.432;0.625)	0.624 (0.524;0.743)	1.098 (0.866;1.393)	0.914 (0.727;1.149)	0.833 (0.657;1.056)
	≥36 mo	306		0.283(0.1158;−0.507)	0.387 (0.230;0.654)	0.300 (0.172;0.523)	0.730 (0.342;1.560)	0.942 (0.430;2.066)	1.291 (0.614;2.713)
Sex	Male	1,078	0.406	0.561 (0.446;0.706)	0.458 (0.354;0.593)	0.555 (0.441;0.700)	1.224 (0.889;1.686)	1.010 (0.750;1.361)	0.825 (0.599;1.137)
	Female	1,078		0.481 (0.377;0.613)	0.535 (0.422;0.678)	0.577 (0.454;0.733)	0.898 (0.650;1.242)	0.833 (0.602;1.152)	0.927 (0.673;1.275)
Breastfeeding at enrolment	No	983	0.755	0.378 (0.286;0.500)	0.394 (0.297;0.523)	0.447 (0.340;0.589)	0.958 (0.659;1.393)	0.845 (0.584;1.221)	0.882 (0.610;1.275)
	Partial	1,173		0.660 (0.537;0.812)	0.592 (0.474;0.739)	0.671 (0.545;0.828)	1.115 (0.838;1.484)	0.983 (0.748;1.292)	0.882 (0.663;1.173)
Study period	Mostly in dry season	1,054	0.320	0.520 (0.413;0.654)	0.453 (0.350;0.585)	0.466 (0.360;0.603)	1.148 (0.829;1.590)	1.115 (0.805;1.544)	0.972 (0.689;1.371)
	Mostly in wet season	1,102		0.529 (0.415;0.673)	0.540 (0.426;0.686)	0.667 (0.536;0.830)	0.978 (0.713;1.342)	0.792 (0.586;1.070)	0.810 (0.600;1.093
Study disease	Only diarrhoea^2^	1,171	0.920	0.637 (0.518;0.783)	0.568 (0.449;0.719)	0.700 (0.567;0.864)	1.121 (0.838;1.502)	0.910 (0.693;1.196)	0.812 (0.605;0.090)
	Only LRTI	127		0.344 (0.145;0.811)	0.317 (0.139;0.720)	0.331 (0.148;0.739)	1.085 (0.347;3.390)	1.037 (0.349;3.087)	0.957 (0.319;2.868)
	Only malaria	314		0.280 (0.157;0.501)	0.404 (0.256;0.638)	0.337 (0.190;0.597)	0.693 (0.347;1.387)	0.832 (0.376;1.841)	1.200 (0.588;2.448)
	≥2 diseases	544		0.482 (0.341;0.682)	0.483 (0.341;0.684)	0.509 (0.363;0.714)	0.999 (0.624;1.597)	0.947 (0.599;1.498)	0.948 (0.606;1.485)
Days of illness before enrolment	1 or 2	377	0.516	0.367 (0.237;0.569)	0.464 (0.307;0.699)	0.453 (0.292;0.703)	0.792 (0.452;1.387)	0.810 (0.457;1.435)	1.023 (0.587;1.784)
	3	752		0.539 (0.407;0.716)	0.395 (0.285;0.549)	0.569 (0.429;0.755)	1.364 (0.908;2.049)	0.948 (0.653;1.378)	0.695 (0.462;1.044)
	≥4	1,027		0.574 (0.455;0.725)	0.585 (0.461;0.743)	0.603 (0.478;0.762)	0.981 (0.717;1.342)	0.952 (0.699;1.295)	0.970 (0.710;1.325)

LRTI: lower respiratory tract infection; MNP: micronutrient powder; RUTF: ready-to-use therapeutic food.

^1^ *p* for interaction between subgroups on differences between arms.

^2^Nonbloody or bloody diarrhoea.

This is despite the fact that in the control group, some subgroups (e.g., partially breast-fed, children younger than 36 mo, those enrolled in the wet seasons) did appear to be at higher risk of developing a NNO (Table 5).

The supplements did not have an effect at 6 mo follow up on other anthropometric indicators such as the average weight gain rate, change in MUAC, change in height, or change in weight-for-age, weight-for-height, and height-for-age of participants who completed the study.

In the first 14 d of the study, no difference in anthropometric indicators was seen between the study groups. However, regardless of the study group, the rate of weight gain was higher in the first 14 d after enrollment compared with the entire study period (for the RUTF, MNP, and control groups: 1.57, 1.47, and 1.50 g/kg/d, respectively, over the first 14 d and 0.87, 0.89, and 0.86 g/kg/d over the entire study period). Similarly, the change in MUAC was higher in the first 14 d compared with the entire study period (for the RUTF, MNP, and control groups: 0.056, 0.055, and 0.039 mm/d, respectively, over the first 14 d and 0.035, 0.036, and 0.033 mm/d over the entire study period) (Table 6).

**Table 6:** Anthropometric indicators: change from baseline to day 14 and day 168.

Indicator		Mean (95%CI)			Mean difference/OR (95%CI)			*p*-Value		
	*n*	RUTF	MNP	Control	RUTF versus MNP	RUTF versus Control	MNP versus Control	RUTF versus MNP	RUTF versus Control	MNP versus Control
**Day 0–day 14**										
Mean rate weight gain (g/kg/d)^1^ ^,^ ^2^	2,025	1.57 (1.33;1.80)	1.47 (1.23;1.71)	1.50 (1.26;1.74)	0.10 (−0.22;0.42)	0.07 (−0.25;0.38)	−0.03 (−0.35;0.29)	0.552	0.686	0.848
Not gaining weight (%)^1^	2,025	58% (47%;68%)	57% (46%;68%)	59% (48%;70%)	0.98 (0.78;1.24)	0.94 (0.75;1.18)	0.93 (0.74;1.17)	0.887	0.524	0.614
Mean MUAC gain (mm/d)^1^ ^,^ ^3^	2,025	0.055 (0.037;0.072)	0.052 (0.035;0.070)	0.039 (0.022;0.056)	0.002 (−0.021;0.025)	0.016 (−0.007;0.038)	0.014 (−0.009;0.037)	0.855	0.173	0.247
**Day 0–day 168**										
Rate weight gain (g/kg/d)^1^ ^,^ ^2^	1,801	0.87 (0.83;0.92)	0.89 (0.85;0.93)	0.86 (0.82;0.90)	−0.02 (−0.07;0.04)	0.01 (−0.04;0.07)	0.03 (−0.03;0.09)	0.580	0.633	0.313
MUAC change rate (mm/d)^1^ ^,^ ^3^	1,801	0.035 (0.031;0.038)	0.034 (0.031;0.038)	0.032 (0.029;0.035)	0.000 (−0.004;0.004)	0.003(−0.002;0007)	0.002 (−0.002;0.006)	0.917	0.229	0.282
Height change (cm)^1^ ^,^ ^4^	1,800	1.112 (1.047;1.177)	1.134 (1.067;1.202)	1.139 (1.072;1.206)	−0.022 (−0.109;0.064)	−0.027 (−0.114;0.059)	−0.005 (−0.093;0.084)	0.612	0.537	0.914
Weight/age change (z-scores)^1^ ^,^ ^2^	1,795	0.251 (0.16;0.27)	0.25 (0.19;0.30)	0.21 (0.15;0.26)	−0.03 (−0.10;0.04)	0.01 (−0.06;0.08)	0.04 (−0.03;0.11)	0.375	0.821	0.276
Height/age change (z-scores)^1^ ^,^ ^4^	1,796	−1.28 (−1.33;-1.22)	−1.22 (−1.28;-1.17)	−1.25 (−1.31;−1.19)	−0.06 (−0.13;0.02)	−0.03(−0.10;0.05)	0.03 (−0.05;0.10)	0.137	0.482	0.444
Weight/height change (z-scores) ^1^ ^,^ ^4^	1,794	1.22 (1.15;1.29)	1.24 (1.16;1.31)	1.19 (1.12;1.27)	−0.02 (−0.11;0.08)	0.03 (−0.07;0.12)	0.05 (−0.05;0.14)	0.713	0.559	0.351

RUTF: Ready To Use Therapeutic Food; MNP: Multi Nutrient Powder; MUAC: Mid Upper Arm Circumference

^1^Adjusted for nutritional status at baseline

^2^Adjusted for weight at baseline

^3^Adjusted for MUAC at baseline

^4^Adjusted for height at baseline.

### Disease and Mortality

More than half of the children experienced one or more new malaria episode (56%, 55%, and 56%, respectively, for the RUTF, MNP, and control groups) during their participation in the study. More than half of the participants had a one or more new LRTI episode (54%, 54%, and 55%, respectively, for RUTF, MNP, and control groups). About three-quarters of participants experienced one or more new episode of acute diarrhoea during the 6-mo follow-up period (79%, 73%, and 78%, respectively, for RUTF, MNP, and control groups). A significantly lower proportion of children in the MNP group had a new diarrhoea episode compared with both the RUTF and control groups (RUTF versus MNP, *p* = 0.007; MNP versus control, *p* = 0.029) (Table 7). The average number of episodes of study diseases during enrollment for the RUTF, MNP, and control groups, respectively, were 4.1, 3.4, and 3.6, with a significant higher number in the RUTF group compared with the MNP and control groups (RUTF versus control, *p* < 0.001; RUTF versus MNP, *p* < 0.001)

**Table 7:** Mean number of diagnosed study diseases and proportion of children with at least one newly diagnosed disease episode or mortality^1^.

Disease	Mean/proportion (95%CI)			Mean difference/OR (95%CI)			*p*-Value		
	RUTF	MNP	Control	RUTF versus MNP	RUTF versus Control	MNP versus Control	RUTF versus MNP	RUTF versus Control	MNP versus Control
	**Number of diagnosed events, mean (95% CI)**			**Mean difference (95%CI)**			***p*-Value**		
Malaria	0.93 (0.84;1.02)	1.05 (0.96;1.14)	0.97 (0.88;1.06)	0.12 (0.00;0.24)	0.08 (−0.04;0.20)	−0.04 (−0.16;0.08)	0.051	0.210	0.484
LRTI	0.92 (0.83;1.01)	0.91 (0.82;1.00)	0.93 (0.84;1.02)	0.02 (−0.10;0.13)	−0.01 (−0.12;0.11)	−0.02 (−0.14;0.10)	0.801	0.921	0.727
Diarrhoea^2^	2.18 (2.05;2.30)	1.55 (1.42;1.68)	1.65 (1.52;1.77)	0.63 (0.46;0.80)	0.53 (0.36;0.70)	−0.10 (−0.27;0.08)	<0.001	<0.001	0.269
All 3 study diseases	4.15 (3.96;4.34)	3.38 (3.19;3.57)	3.55 (3.36;3.74)	0.76 (0.51;1.02)	0.60 (0.35;0.85)	−0.16 (−0.42;0.09)	<0.001	<0.001	0.215
	**Proportion of participants having at least one new disease or death (95% CI)**			**Odds Ratio** ^**3**^ **(95% CI)**			***p*-Value**		
Malaria	56%(52;60)	55% (51;59)	56% (52;60)	1.04 (0.85;1.28)	1.00 (0.81;1.23)	0.96 (0.78;1.18)	0.680	0.985	0.669
LRTI	54%(50;58)	54% (50;58)	55% (51;59)	1.01 (0.82;1.23)	0.98 (0.80;1.20)	0.97(0.79;1.20)	0.948	0.839	0.791
Diarrhoea^2^	79% (76;82)	73%(69;76)	78%(75;81)	1.41 (1.10;1.79)	1.07 (0.83;1.38)	0.76 (0.60;0.97)	0.007	0.604	0.029
Mortality^1^	0.8%(0.3;1.8)	1.8% (1.0;3.3)	1.4% (0.7;2.8)	0.42 (0.16;1.12)	0.55 (0.20;1.53)	1.31 (0.57;3.00)	0.084	0.254	0.531

LRTI: lower respiratory tract infection; MNP: micronutrient powder; RUTF: ready-to-use therapeutic food.

^1^ *n* = 2,156, but for mortality *n* = 1,829 (in participants with known outcome).

^2^Nonbloody or bloody diarrhoea.

^3^Adjusted for nutritional status at baseline.

A total of 29 (1.3%) children died during the study: in the RUTF, MNP, and control groups, respectively, 6, 13, and 10 children died (RUTF versus MNP, *p* = 0.084). The causes of death could be related to malaria (17; 58.6%), diarrhoea and dehydration (9, 31.0%) and LRTI or sepsis (3, 10.3%).

The number of patients who died from malaria in the RUTF, MNP, and control groups, respectively, was 3 (0.4%), 8 (1.1%), and 6 (0.8%); for diarrhoea, the numbers were 2 (0.3%), 4 (0.6%), and 3 (0.4%); and for LRTI, 1 (0.1%) death occurred in each intervention group. Of the 29 deceased children, 17 died at home, 7 at the hospital, 4 while hospitalised in the therapeutic feeding programme, and 1 on arrival at the hospital. In all, 54 participants needed to be admitted to hospital (21, 20, and 13 children in the RUTF, MNP, and control groups, respectively). The main cause of admission was malaria, followed by diarrhoea. The number of patients admitted due to malaria was 9 (1.2%), 10 (1.4%), and 8 (1.1%), respectively, for the RUTF, MNP, and control groups; the numbers for diarrhoea were 5 (0.7%), 7 (1.0%), and 1 (0.1%); and for LRTI they were 5 (0.7%), 3 (0.4%), and 2 (0.3%).

Causes of death and causes of hospital admissions did not show a statistically significant difference between the study arms, even when mortality and hospital admission were combined, although most admissions and deaths related to malaria and diarrhoea were in the MNP group (Table 8).

**Table 8:** Mortality and hospital admissions by intervention group.

Event		RUTF (*n* = 739)		MNP (*n* = 717)		Control (*n* = 732)		Total (*n* = 2,188)	
**Deaths**									
Total deaths		6	0.8%	13	1.8%	10	1.3%	29	1.3%
Cause	Malaria	3	0.4%	8	1.1%	6	0.8%	17	0.8%
	LRTI	1	0.1%	1	0.1%	1	0.1%	3	0.1%
	Diarrhoea/dehydration	2	0.3%	4	0.6%	3	0.4%	9	0.4%
**Hospitalizations**									
Total hospitalizations		21	2.8%	20	2.8%	13	1.7%	54	2.4%
Cause	Malaria	9	1.2%	10	1.4%	8	1.1%	27	1.2%
	LRTI	5	0.7%	3	0.4%	2	0.3%	10	0.5%
	Diarrhoea/dehydration	5	0.7%	7	1.0%	1	0.1%	13	0.6%
	Other^1^	2	0.3%	0	0	2	0.3%	4	0.2%

LRTI: lower respiratory tract infection; MNP: micronutrient powder; RUTF: ready-to-use therapeutic food.

^1^Convulsions, abdominal pain, sepsis, measles (cause of admission unknown in one case).

### Consumption and Compliance

Supplement consumption over the first 14 d after enrollment as reported by the caretaker showed that participants in the RUTF group consumed an average of 13.8 sachets (per allocation of 14 sachets) and participants in the MNP group consumed 26.9 sachets (per allocation of 28 sachets). Over the entire study period and all allocations, an average of 13.8 sachets of RUTF (out of 14 sachets) was consumed and 27.0 sachets of MNP (out of 28 sachets) (Table 9).

**Table 9:** Allocation to and compliance with supplements in intervention group.

Measure	RUTF		MNP		Control	
	Day 0–14	Study period	Day 0–14	Study period	Day 0–14	Study period
Number of allocations assigned	739	2,324	717	2,026	732	2,096
Number of consumption reports (*n*, % of allocations)	693 (93.8%)	1799 (77.4%)	676 (94.3%)	1,549 (76.5%)		
Number sachets consumed (mean ± SD)	13.8 (±0.8)	13.8 (±0.7)	26.9 (±3.6)	27.0 (±3.0)		
Consumed 14 for RUTF or 28 for MNP (*n*, % of allocations)	649 (93.7%)	1,720 (95.6%)	586 (86.7%)	1,364 (88.2%)		

MNP: micronutrient powder; RUTF: ready-to-use therapeutic food.

Focus group discussions showed that all the mothers enjoyed giving RUTF or MNP to their children. About RUTF, they mentioned the following benefits: “Improvement of health, increased appetite, more powerful, gain the body weight.”In addition, MNP was reported to increase appetite, give more stamina, make the child stronger, and reduce diseases. The mothers expressed wanting to buy RUTF but also MNP if the price was reasonable, as they perceived it as improving their child’s health and increasing appetite and weight. Mothers reported reserving the supplement for the study child, as they saw it as part of the treatment for the disease.

In both groups, all mothers found the instructions on how to administer the supplement clear and easy to follow. The malaria treatment was also discussed; mothers said they now know how to give the medication and how important it is to finish the treatment.

## Discussion

### Incidence of Malnutrition

This study and its companion trial in Kaabong (Uganda) are the first large randomised controlled trials of the use of novel supplements (RUTF and MNP) for the prevention of malnutrition in children with a nonsevere illness. In our site in Nigeria with a high incidence of disease, there was no reduction in incidence of malnutrition with short-term supplementation of either RUTF or MNP compared with a control group. Supplementation had no impact on any of the anthropometric indices, and no effect of supplementation was found when comparing specific subgroups. The MNP group showed a lower number of events for diarrhoea but had a higher number of malaria events compared with the RUTF and control groups. There were fewer deaths in the RUTF group than in the control and MNP groups, this did not achieve statistical significance.

In comparison, a similar study in Kaabong (Uganda) found a significant reduction in the incidence of malnutrition in the RUTF group, but not in the MNP group (0.143, 0.185, and 0.213 first events/y for RUTF, MNP, and control, respectively; RUTF versus control, *p* = 0.037) [30].

We also found an overall high incidence of malnutrition in Goronyo; it was higher than anticipated at the beginning of the study. The sample size of the study was based on an incidence of first time NNO in the control group of 0.435 events/y, but the study showed 0.566 events/y in the control group, suggesting that the incidence of malnutrition among previously ill children in Goronyo was underestimated. In comparison, the incidence of malnutrition in the control group in Kaabong was only 0.213 events/y, which is low [30]. However, the study in Kaabong did not include moderately malnourished children, but the incidence of malnutrition among those who were non-malnourished in Goronyo was still high with 0.581 events/y. The incidence in Goronyo was also higher than the 0.26 events/year found in a study among the general population of children (not necessarily having been ill) in Niger [22]. This suggests that the incidence of malnutrition in Goronyo is higher than that of other countries. Goronyo is not the poorest area compared with the other surrounding countries, so other factors than poverty are likely to influence the incidence of malnutrition.

The incidence rate of severe malnutrition among participants who were moderately malnourished upon enrollment was high: a third developed severe malnutrition in the study period (incidence 0.32/168 d). The extrapolated incidence rate of 0.711/y might be misleading as it is unknown whether initial moderately malnourished children who did not develop severe malnutrition in during the first 6 mo will do so in the following 6 mo. Nevertheless, treatment of moderate malnutrition in ill children will prevent for a large part life threatening severe malnutrition in this group.

We expected that supplementation would be more effective among moderately malnourished children, as their nutritional reserves are already marginal, and supplementation could provide just the nutritional support needed. However, our study showed that supplementation was not effective in reduction of severe malnutrition in moderately malnourished ill children.

The lack of effectiveness of supplementation among moderate malnourished children could be due to the period of supplementation, which may have been too short, as moderately malnourished children need supplementation for at least 4 wk and often longer before being cured from malnutrition. Alternatively, the dose of the supplements may have been too low to be effective in supporting nutritional recovery in illness in moderately malnourished children, specifically when the morbidity is high. Finally, a potential effect of supplementation might be overshadowed by the high morbidity in Goronyo, resulting in a net loss of nutrients despite supplementation, which may be more important in the moderately malnourished group.

### Morbidity and Mortality

The mean number of newly diagnosed study diseases was high. In total, more than 70% of participants reported at least one new diarrhoea episode, more than 50% a new LRTI episode, and more than 50% a new malaria episode. In total, the average number of new study diseases in the control group was 3.6 new events during the study period for the three study diseases together. In the study in Kaabong, the average number of new study diseases was 2.3 new events for the three study diseases together [30], which is lower than in Goronyo. When the illness on enrollment is included, this means that a child had an average of 4.6 disease episodes in 6 mo, which is about one illness every one and a half months.

Caretakers waited an average of 4.5 d (2.5 d in Kaabong) before presenting at a clinic when a child fell ill (as measured on enrollment). A delay in seeking treatment for diseases makes children more vulnerable to malnutrition, and in this study we found that the incidence of NNO was higher when the mothers waited longer before visiting the clinic.

The high morbidity in Goronyo is also reflected in the hospital admission and mortality rate. Fifty-five (2.5%) participants spent at least one night in the hospital, and 29 (1.3%) children died during the study. The study is unlikely to have underestimated mortality, as all participants who stopped attending the study were followed up and information from the family and their neighbours was obtained on the whereabouts of the child. The RUTF group showed the lowest mortality, and this was also observed in the Kaabong study, with no deaths in the RUTF group [30]. Our results are similar to those of a cluster randomised trial in Niger that showed a nonsignificant lower mortality in the intervention group (adjusted hazard ratio, 0.51; 95% CI, 0.25–1.05) [22]. These findings, while inconclusive, merit further research, as does the finding that the mortality was highest in the MNP group in both trial sites.

These findings show that the disease burden, the frequency of illnesses, and the delay in treatment in Goronyo was high. This could also be a reason why a supplement of short duration did not show an effect on the incidence of malnutrition. While a usual convalescence time from a disease would be at most 2 weeks, as treatment often lasts 1–2 days for diarrhoea, 3 d for malaria, and 5 d for LRTI. In addition, more than one-third of the participants had difficulties with gaining weight in the 2 wk after their illness. This lack of improvement indicates that recovery from illness was difficult. A supplementation period of 2 wk may not be enough to mitigate weight loss due to the frequent episodes of illness in Goronyo.

We compared Goronyo with other studies that supplemented all children in a population (not necessarily ill), as there is a lack of studies using modern supplements in ill children. However, this assumes that all children in the population are at equal risk of a high morbidity, and that the group of ill children in our study are comparable with all children in the population.

A longer supplementation period was effective in a study in Burkina Faso, with participants having also a considerable morbidity burden. In this study, a small quantity of LNS (with several levels of zinc) alongside morbidity surveillance and treatment and nutrition education was provided for 9 mo to children aged 9 mo. This intervention had a significant effect on all anthropometric indicators including wasting, which was 8.7% in the intervention group and 13.5% in the control group [23]. The control group did not receive morbidity surveillance and treatment, and the combination of medical care and supplementation could be essential for the significant reduction in incidence of malnutrition in Burkina Faso in the intervention group [23]. A study in Chad, where a small quantity of LNS was provided for 4 mo alongside a general food distribution, but where no specific element of treatment of morbidity was added, did not show a reduction of incidence of wasting [24]. In contrast, a research in Niger providing RUTF and morbidity surveillance and treatment for 4 months did show a significant reduction in wasting of 36% [22]. This indicates that a high quality supplement for a longer period in combination with morbidity surveillance and treatment is more effective in reducing the incidence of acute malnutrition in contexts with a high burden of morbidity then only providing a nutritional supplement.

### Risks

There are reports suggesting an increased risk of severe illness when supplementing children with micronutrients. Soofi et al. [19] found a significant increase in rapid breathing and chest in-drawing in their MNP group (including zinc); however, this concern is not confirmed by our findings, as the rate of hospital admission or death due to LRTI or sepsis was low and not significantly different between the study groups, despite the fact that we were using twice the recommended dose of supplementation for MNP.

In our study, we used two doses of micronutrients resulting in twice the amount of recommended iron of 20 mg instead of 10 mg per day. Literature has suggested that iron supplements might worsen the outcome of malaria episodes [37] [38]. A review that was published before the start of our study and a later review did not confirm negative effects of iron supplementation on the outcome of malaria [39,40]. The mean number of diagnosed malaria events was higher in MNP group than in the RUTF group (*p* = 0.051). The mean number of morbidity events is not necessarily a new event; a child may still be ill with a previously diagnosed illness. A more robust indicator is the proportion of children with at least one new malaria episode, which as similar for all intervention groups. Also the number of hospital admissions and deaths due to malaria was higher (not significant) for MNP group compared with RUTF group. There was no difference in severe outcome between MNP and the control group; therefore, it is also possible that RUTF reduces the risk instead of MNP increasing the risk of a severe outcome.

Soofi et al. [19] also found an increased risk for severe diarrhoea and complications in their MNP groups. In contrast to their findings, in our study the MNP group had a significantly lower proportion of children having diarrhoea (non-severe) compared with the other two study groups. However, of the participants with severe diarrhoea or dehydration requiring hospital admission or resulting in death, a higher proportion came from the MNP group than the RUTF and control groups, but the numbers were small.

Overall, it seems that MNP shows a higher risk of severe disease and consequent mortality compared with RUTF but for MNP compared with the control group this is less clear. Moreover, it is also possible that RUTF lowers the risk of severe illness and outcome including severe malaria and severe diarrhoea/dehydration, which supports the observations that RUTF or lipid-based nutrient supplementation reduces mortality, as found in several studies [22,30]. As the numbers of hospital admissions and deaths were low, we cannot conclude or confirm an increased risk of micronutrient supplementation in ill children who are properly treated for their diseases.

### Limitations

Despite the robust study protocols and strict implementation, this study had some limitations. First of all, the participants were not blinded for the RUTF or MNP supplementation. As the population had a positive attitude towards RUTF, this could introduce a bias to more positive reporting and compliance and a lower lost to follow up compared with MNP. However, blinding is difficult to achieve for RUTF, and efforts to produce a placebo for MNP failed. We think that, because MNP was new to the population, there were no positive or negative feelings about the product prior to participating in the study. The focus group discussions revealed that both RUTF and MNP were associated with positive health effects by the caregivers.

The sampling methodology was simple randomisation. This cannot exclude a spill-over effect of the different supplementation groups, as children in different households live relatively near to each other. Specifically, the RUTF could have been consumed by children in non-RUTF arms. It was not possible to adapt the sampling methodology towards a cluster randomised design owing to security considerations.

Diarrhoea was the only study disease that was based on caretakers’ report. As RUTF was popular among the caretakers, it is possible that participants reported diarrhoea more often in the hope of receiving RUTF; over-reporting of diarrhoea in the RUTF group cannot therefore be ruled out.

A lower compliance than measured cannot be ruled out despite the fact that the measured compliance and information from the focus group discussions suggested a high compliance and the analysis of the PP population did not give a different result. Abbeddou et al. found when doing 12-h observations in households that about 50% of supplements would be given, while measurement of compliance by questionnaires and counting of returned wrappings gave a much higher compliance [41]. This suggests that we cannot exclude a lower compliance than reported by the caretakers.

The results cannot be generalised to the general population of children. Although the participants were often ill, it cannot be assumed that all children in the population are often ill and thus that the result would apply to all children. Theoretically, it is possible that another group of children in the general population is hardly ever ill. Therefore, comparison with research implemented in the general population of children should be interpreted with caution.

### Conclusions

In this randomised controlled trial of two-week supplementation with RUTF or MNP to ill children as part of routine primary medical care to children with malaria, pneumonia, or diarrhoea, we were unable to show a reduction in incidence of malnutrition. The lack of effect in Goronyo may be due to a high frequency of morbidity, which probably further affects a child’s nutritional status and children’s ability to escape from the illness–malnutrition cycle. The duration of the supplementation may have been too short or the doses of the supplements may have been too low to mitigate the effects of high morbidity and pre-existing malnutrition. An integrated approach combining prevention and treatment of diseases and treatment of moderate malnutrition, rather than prevention of malnutrition by nutritional supplementation alone, might be more effective in reducing the incidence of acute malnutrition in ill children. Further research should clarify the most cost-effective integrated approach, specifically in areas with high morbidity. Also, the extent LNS such as RUTF contributes to a lower mortality should be investigated.

## Supporting Information

S1 Text Protocol. (PDF)

S2 Text Statistical Analysis Plan (SAP). (PDF)

S3 Text CONSORT Checklist. (PDF)

S1 Data Line list endpoints. (TXT)

## Abbreviations

DRC: Democratic Republic of Congo
GAM: global acute malnutrition
ITT: intention to treat
LRTI: lower respiratory tract infection
LNS: lipid-based nutrient supplements
MAM: moderate acute malnutrition
MNP: micronutrient powder
MSF: Médecins Sans Frontières
MSF-OCA: Médecins sans Frontières-Operational Centre Amsterdam
MUAC: mid-upper arm circumference
NNO: negative nutritional outcome
PP: per protocol
RUTF: ready-to-use therapeutic food
SAM: severe acute malnutrition
SAP: statistical analysis plan
TFC: therapeutic feeding centre
WHO: World Health Organization
