# Supplementary material for: Effect of Short-Term Supplementation with Ready-to-Use Therapeutic Food or Micronutrients for Children after Illness for Prevention of Malnutrition: A Randomised Controlled Trial in Nigeria
Source: PLoS Med. 2016 Feb 9;13(2):e1001952. doi: 10.1371/journal.pmed.1001952 (PMC4747530; doi:10.1371/journal.pmed.1001952)
Supplement: S1 Text — (PDF) [file pmed.1001952.s001.pdf]

STUDY PROTOCOL

Effectiveness of nutritional supplementation (RUTF and multi micronutrient) in preventing malnutrition in children 6-59 months with infection (malaria, pneumonia, diarrhoea), a randomized controlled trial in Nigeria

Principle Investigator: Kam, S van der Ir
Contributors: Swarthout TD. MPH, Shanks L. MD, Jiya N.M. MD, Boele-van Hensbroek M. MD PhD, Gómez Restrepo C. MD, Roll S. Dr PhD, Tinnemann P. MD MPH

Affiliations:
- Kam, S van der : Nutritionist, Médecins Sans Frontières, Amsterdam
- Swarthout TD: Epidemiologist, Médecins Sans Frontières, Amsterdam
- Field Research Coodinator: Alyson Froud, Médecins Sans Frontières, Amsterdam
- Shanks L: Medical Director, Médecins Sans Frontières Amsterdam
- Jiya NM: Paediatrician, Usman Danfodio University Teaching Hospital (UDUTH), Sokoto, Nigeria
- Boele van Hensbroek M: Peadiatrician, Global Child Health Group, EKZ-AMC
- Gómez Restrepo C: Medical coordinator in Nigeria, Médecins Sans Frontières, Amsterdam
- Roll S: Statistician, Institute for Social medicine, Epidemiology and Health Economics, Charité University Medical Center, Berlin, Germany
- Tinnemann P: Scientist, Institute for Social medicine, Epidemiology and Health Economics, Charité University Medical Centre, Berlin, Germany

Contact:
Saskia van der Kam
Médecins Sans Frontières Holland
Plantage Middenlaan 14
1018 DD Amsterdam
tel: 00 31 20 5208 978
Saskia.vd.kam@amsterdam.msf.org

Registered:
www.clinicaltrials.gov
Number NCT01154803

Abbreviations

AE	Adverse event
AR	Adverse reaction
DOTS	Directly observed treatment
DRC	Democratic Republic of Congo
EDTA	Ethylene diamine tetra-acetate
FRC	Field research coordinator
GAM	Global acute malnutrition
HQ	Headquarters
ITT	Intention to treat
LGA	Local Government Authority
LRTI	Lower respiratory tract infection
MAM	Moderate acute malnutrition
MCH	Mother and child health
MoH	Ministry of Health
MMN	Multi-micronutrient
MNP	Multi-micronutrient powder
MSF-OCA	Médecins Sans Frontières – Operational Centre Amsterdam
MUAC	Mid upper arm circumference
NNO	Negative nutritional outcome
OPD	Outpatient department
PI	Principle investigator
PP	Per protocol
QC	Quality control
RDT	Rapid diagnostic test
RDI	Recommended daily intake
RNI	Recommended nutritional intake
RR	Risk ratio
RUF	Ready to use food
RUTF	Ready to use therapeutic food
SAE	Serious adverse event
SAM	Severe acute malnutrition
SAP	Statistical analysis plan
SAR	Serious adverse reaction
TFP	Therapeutic feeding program
U5MR	Under-five mortality rate
UNICEF	United Nations of International Children's Emergency Fund
W/H	Weight-for-height
WFP	World Food Programme
WHO	World Health Organization

Glossary

Not malnourished	W/H ≥ -2 z-scores
Moderate acute malnutrition	W/H < -2 and ≥ -3 z-scores
Severe acute malnutrition (SAM)	W/H < -3 z-scores or<br>MUAC < 115 or<br>Bilateral oedema
Acute malnutrition:	Includes either SAM or MAM
Global acute malnutrition (GAM)	W/H < -2 z-scores or<br>MUAC < 115 or<br>Bilateral oedema
Rate of weight change	Grams weight change / kg body weight at inclusion / days follow-up

Table of contents

Abbreviations 2
Glossary 3
Table of contents 4
SUMMARY 6
1. INTRODUCTION 8
1.1 Background 8
1.2 The inter-relationship between infection and malnutrition 8
1.3 Effect of supplementation 8
1.3.1 Supplementation to prevent malnutrition 8
1.3.2 Supplementation and reduced risk of disease 9
1.4 Supplements 9
1.4.1 Ready to Use Therapeutic Food 9
1.4.2 Multi-Micronutrient powder 9
1.5 Rationale for the study 10
2. STUDY OBJECTIVES 11
3. STUDY DESIGN 12
4. STUDY SITE 12
4.1 Number of Participants 12
4.2 Inclusion criteria 12
4.3 Exclusion criteria 12
5. PARTICIPANT SELECTION AND ENROLMENT 13
5.1 Identifying participants 13
5.2 Consenting participants 13
5.3 Screening for eligibility 13
5.4 Ineligible and non-recruited participants 13
6. RANDOMISATION 13
6.1 Randomisation 13
6.2 Blinding 14
6.3 Treatment allocation 14
7. NUTRITIONAL SUPPLEMENTATION 14
7.1 RUTF (Plumpynut®) 14
7.2 MNP (MixMe®) 14
7.3 Control group 14
8. CLINICAL PROCEDURES AND METHODOLOGY 15
8.1 Study clinic procedures at day of inclusion (day-0) 15
8.2 Follow-up schedule 15
8.3 Measuring malnutrition 16
8.3.1 Weight 16
8.3.2 Height 17
8.3.3 MUAC 17
8.4 Three study diseases 17
8.5 Compliance 17
8.6 Emergency unblinding procedures 17
8.7 Withdrawal procedures 17
8.8 Endpoints 18
8.8.1 Primary endpoint 18
8.8.2 Secondary endpoints 18
8.9 Data collection 18
9. DATA ANALYSIS 19

9.1 Sample size 19
9.2 Populations for analysis 19
9.3 Descriptive Analyses 20
9.3.1 Primary analysis of the primary endpoint 20
9.3.2 Secondary analysis of the primary endpoint 20
9.3.3 Analyses of secondary endpoints 22
9.3.4 Pooled analysis 22
9.3.5 Statistical analysis plan and software 22
10. QUALITY CONTROL 22
10.1 Coordination 22
10.2 Diagnostics 22
10.3 Staff, Training, and Supervision 22
11. ETHICAL CONSIDERATIONS 22
11.1 Treatment of participants 22
11.2 Cooperation with national and local partners 23
11.3 Benefits to the community 23
11.4 Feedback of results 24
11.5 Potential risks 24
11.6 Adverse events 24
11.7 Interim analysis 25
12. STUDY INVESTIGATORS AND THEIR ROLES 25
13. REFERENCES 28

SUMMARY

Title:
Effectiveness of nutritional supplementation (RUTF and multi micronutrient) in preventing malnutrition in children 6-59 months with infection (malaria, pneumonia, diarrhoea), a randomized controlled trial in Nigeria

Study investigators
PI: van der Kam, S. Ir
Epidemiologist: Swarthout, TD. MPH
Field study coordinator: not recruited yet
Statistician: Roll, S. Dr, PhD
Paediatrician Nigeria: N. M. MD.
Paediatrician: Boele van Hensbroek, M. MD PhD
Medical responsible MSF Nigeria: Gómez Restrepo, C. MD
Institutional responsible MSF: Shanks, L. MD
Academic advisor: Tinnemann, P. MD MPH

Status: Protocol with Ethical Review Board.
Start date: 30th August 2010.
Location: Outpatient Department (OPD) of the health clinic in Goronyo LGA, Sokoto State, Nigeria.

Objectives
Overall objective: To determine the effectiveness of 14 days supplementation with Ready to use therapeutic Food (RUTF) or micronutrients alone concurrently with treatment for diarrhoea, malaria or lower respiratory tract infection (LRTI) in reducing risk of malnutrition and disease.

1. ***Primary aim:*** Effectiveness of supplementation with RUTF concurrently with treatment for diarrhoea, malaria or LRTI in reducing incidence of malnutrition
2. ***Secondary aims:***
    a. Effectiveness of supplementation with a multi-micronutrient powder (MNP) concurrently with treatment for diarrhoea, malaria or LRTI in reducing incidence of malnutrition
    b. Effectiveness of supplementation with RUTF or MNP on reduction of frequency of diarrhoea, malaria or LRTI
    c. Explore effectiveness of the supplements in reducing malnutrition and illness for moderate malnourished, young children and breastfed children

Participant population
Children 6 months to 59 months of age presenting at the OPD with diarrhoea, malaria or LRTI.

Inclusion criteria
- 6 to 59 months of age.
- Non-acutely malnourished or moderately acutely malnourished children.
- Diagnosis of malaria and/or diarrhoea and/or LRTI.
- Intention to remain in area for the duration of the 6 month follow-up.
- Lives within approximately 60 minutes walking distance from the clinic.
- Informed consent from a guardian.

Exclusion criteria

- Child is exclusively breastfeeding.
- Severe acute malnourishment of child.
- Presence of 'General Danger Signs'.
- Presence of severe disease (incl. severe malaria, severe LRTI, severe diarrhoea).
- Need of hospitalisation for any reason.
- Known history of allergy to the nutritional supplementation
- Sibling already enrolled in the study

Summary of study design

This is a partially blinded randomized controlled trial with three study groups each with 734 participants.

Children participating in this study will be randomised to one of 3 study groups to
A) Receive 14 days of RUTF supplementation with standard care and treatment or
B) Receive 14 days of Multi-micronutrient powder (MNP) supplementation with standard care and treatment or
C) Be included in a control group receiving standard care and treatment but not receiving nutritional supplementation
Individual follow-up will be 6 months. During this time, children included in the RUTF or MNP group will receive 14 days nutritional supplement every time diagnosed with at least one of the three study diseases, not exceeding more than 14 days supplementation in any 28 day period. Children randomly assigned to the control group will receive equivalent care and follow-up as children receiving supplementation. The only difference is that they will not receive a nutritional supplement. Current clinical practice does not include nutritional supplement.

At recruitment written consent of the guardian is obtained. Baseline information will be collected and medical examination will be performed.
After 14 days and 28 days, and thereafter every month, the participant will be weighed and measured, medically checked and questioned about illness.

Accrual Goals

To have baseline, monthly and end data of weight and morbidity from at least 660 participants per group.

Progress to date

- The protocol is written including the tools and questionnaires.
- Ethical review is sought with MSF and The Nigerian ethical review committee.
- The RUTF and micronutrients are available
- The field investigator is being recruited.
- Foreseen start date of preparation in the field: 15th November 2010.
- Start date of recruitment: December 2010.

1. INTRODUCTION

1.1 Background

The global burden of malnutrition is staggering, with an estimated 10% prevalence of moderate acute malnutrition (55 million children) and 3.5% severe acute malnutrition (19 million children). [1] These malnourished children have a higher risk of mortality, ranging from a 3-fold increased risk for the moderately malnourished to a nearly 10-fold increase for the severely malnourished.

In sub-Saharan Africa malnutrition occurs predominantly in children under 3 years of age and is prevalent throughout the year. In many settings food security is often limited and the burden of disease debilitating for families and communities. Though difficult to quantify the risk of poor outcome that malnutrition adds to children who are ill, nutritional supplementation is considered critically important as a means to improve nutritional state and chances of successful convalescence.

In Nigeria, a chronic existence of malnutrition is linked to a marginal economic situation and under resourced health care system. In the Goronyo Local Government Authority (LGA) in Sokoto State, northwest Nigeria, a 2009 survey performed by Médecins Sans Frontières before the hunger season showed a 14.8% prevalence of global acute malnutrition (GAM), and 4.9% severe acute malnutrition (SAM). The prevalence of stunting, a sign of chronic malnutrition, was 57% and there was an under five mortality rate (U5MR) of 1.67/10,000/day. [MSF-OCA, Data not published]

In the same study, among 789 children, 51% reported having an illness at some time during the previous 14 days. Normally this percentage is approximately 25% in resource poor settings , suggesting a potentially high burden of disease that likely exacerbates an already precarious problem of malnutrition. The survey further showed a strong link between morbidity and malnutrition, with 75% and 82% of acutely moderate and severe malnourished children, respectively, reportedly sick in the 14 days prior to the study. Similarly, the prevalence of malnutrition amongst those children who reported an illness in the previous 14 days was 23%, compared to 7% among non sick children ($P<0.001$).

In the Goronyo Local Government Authority (LGA), in collaboration with the local and State Ministry of Health, Médecins Sans Frontières supports the Mother Child Health (MCH) Clinic and operates a nutritional feeding program with two outpatient departments (OPD). Teams work to reduce the two key inter-related factors that contribute to poor health in this community, infection and malnutrition. During 2009, there were 33, 833 new clinical visits for children under 5 years of age; 30% (10,191) included a malaria diagnosis, 21% (7210) diarrhoea and 9% (3045) a lower respiratory tract infection (LRTI).

1.2 The inter-relationship between infection and malnutrition

The cause of malnutrition in most tropical countries is multi-factorial, involving not only an inadequate diet but also recurrent infections [2, 3]. Infections can lead to both anorexia and increased nutritional needs for recovery. These competing needs often lead to weight loss. Work by Rowland *et al.*, for example, showed that amongst children 6-36 months of age, diarrhoea and malaria contributed to a mean weight loss of 746 grams and 1072 grams per episode, respectively [4].

Failure to return to normal nutritional status after an illness increases a child's susceptibility to further infections, perpetuating a cycle towards further reduced nutritional state [4-7]. When inadequate nutrition limits recovery, there is increased risk of a permanently lowered nutritional status [8].

However, there are reports that acute weight loss during episodes of acute infection can be mitigated by good nutrition. [9, 10]

1.3 Effect of supplementation

1.3.1 Supplementation to prevent malnutrition

In order to prevent malnutrition after an infection, the World Health Organisation (WHO) recommends that caretakers give children one additional healthy meal daily during the two weeks after the onset of illness. [11] In resource poor settings this strategy is likely to be inappropriate, as caregivers often lack resources, including healthy ingredients, to implement this recommendation. Therefore, a more

effective strategy in resource poor areas may be to provide an ill child with a nutritional supplement. One option would be to provide this supplement at point of care.

Meta-analyses also report the significant positive effect of multi-micronutrient supplementation on child weight, using pooled data from interventions with a duration ranging from 8 to 52 weeks [12].

Supplementation amongst ill children could also result in improved weight gain. Weight loss during infections is caused primarily by anorexia. Micronutrients play an important role in the regulation of anorexia. An infection can induce secondary micronutrients shortages and a shortage of some micronutrients might cause anorexia to persist over time.
Therefore supplementation of multi-micronutrients might mitigate weight loss in sick children, though evidence is scarce. One study, in The Gambia, showed that weight loss amongst children with acute diarrhoea was significantly lower when provided a supplement enriched with vitamins and minerals for 2 weeks after diagnosis, compared to a control group with diarrhoea but no supplement. [10]

1.3.2 Supplementation and reduced risk of disease

The effect of supplementation in the vicious cycle of malnutrition and diseases could be two fold: reducing weight loss during illness and reducing the frequency of illness.

Several micronutrients have been proven to reduce the severity and frequency of illness. Studies have shown that zinc supplementation during diarrhoea reduces the severity and frequency of both diarrhoea and lower respiratory tract infections.[13] Vitamin A supplementation has been shown to reduce mortality in general. [14]
In the fore mentioned DRC study [15], those receiving supplementation were significantly less likely to report cough at 14 days after beginning the supplementation (AOR: 2.85, P=0.005), suggesting disease can be reduced with supplementation.

1.4 Supplements

Micronutrients can be delivered in a number of forms, including pastes (including energy and proteins) or powders.

1.4.1 Ready to Use Therapeutic Food

Ready to Use Therapeutic Food (RUTF), is a paste and packaged in individual portions that can be eaten directly from the package without additional handling or preparation. There are several brands of RUTF currently available and widely used (e.g. by UNICEF), including Plumpynut® and Eezeepaste®. It is designed for catch up growth in malnourished children [16-18] and mass distribution resulted in prevention of malnutrition. [19]
Therefore RUTF can be expected to promote weight gain after a period of weight loss due to infection.

In the Democratic Republic of Congo (DRC) van der Kam *et al.* studied potential benefits on weight gain after providing RUTF to children 6-59 months of age with a confirmed malaria infection. Children were randomised to receive either 14 days of a RUTF or to receive no nutritional intervention. Results showed that though the intervention group had a significantly greater mean weight gain during the first 14 days (P=0.005). Both groups showed a similar mean weight gain at 28 days of follow-up that can be contributed to the relatively stable food security situation in the study area allowing for catch up growth. [15]
The faster recovery in the supplementation group could be more relevant in food insecure areas with a high disease burden.

1.4.2 Multi-Micronutrient powder

The WHO, WFP and UNICEF specify a recommended micronutrient composition as a supplement for healthy children. When fortified rations are not available, children aged 6 to 59 months should be given a daily dose of the micronutrient supplement (See Annex 11 composition Mixme and Plumpynut). [20].
Multi-micronutrients are can be delivered in the form of a powder. The micronutrient powder (MNP) is tasteless, colourless and are spread over or mixed with a meal prior to consumption. MNPs are packed in very small sachets that contain a quantity appropriate for one meal for one person. Advantages of MNP's include being highly portable (MNPs do not include macronutrients) and their acceptability. The latter is especially important for children who usually show a low acceptability and compliance for tablets. There are two key brands of MNP currently available and widely used (e.g. by WFP and

UNICEF), including MixMe® and Sprinkles®. Both are well tolerated by children and caregivers and are easy to administer resulting in a high compliance. In Bangladesh, compliance is reportedly 80-85%.[21, 22] MNP's have shown to be successful in treating and preventing anaemia. [23, 24]

1.5 Rationale for the study

MSF works throughout the world providing essential medical care through basic health care services, including nutritional feeding programmes and OPD clinics. Given the endemicity and magnitude of illnesses in sub-Saharan Africa, this study aims to explore the effectiveness of nutritional supplementation concurrently with an episode of one of the three key morbidities (malaria, diarrhoea and LRTI) in reducing incidence of malnutrition and recurrence of these three morbidities. If shown to be effective, MSF would consider incorporating nutritional support in its OPD protocols as supportive therapy for these infections and as a routine component of the strategy in preventing malnutrition and recurrent infection. The interventions studied are also felt to have wider relevance outside of MSF, and if results are positive, MSF will advocate to Ministries of Health, WHO and donors to include the intervention in their basic package of care.

To date there has been no evidence to show the impact of nutritional supplementation on incidence of malnutrition or morbidities amongst routine users of OPD services. In addition, there is limited information on how to roll out such an intervention, including which populations to target, the composition suitable for ill children and the preferred form of delivering such supplements.

Though RUTF is a standard fortified food product seen in many MSF interventions, it is a logistically heavy product (i.e. cost, bulk and weight). The question remains as to whether it is essential to include the macronutrient components (incl. energy and proteins) of RUTF. It is not evident that macronutrients are necessary to prevent malnutrition in children with disease, as anorexia is modulated by micronutrients. It is important from a cost effectiveness perspective to investigate whether micronutrients alone would prevent malnutrition in ill children. For this reason, two commonly used supplements will be investigated, i.) an RUTF with both macro- and micro-nutrients and ii.) a powder with only the multi-micronutrient component.

The duration of the supplementation should cover the period to recover from weight loss and disease. A study from Gambia suggests that recovery from weight loss for diarrhoea occurs within the first 14 days [10]. In addition, 14 days will cover convalescence from disease. Finally 14 days is an operationally feasible intervention.

While timely and appropriate treatment of disease is the priority, a nutritional supplement for a short duration (14 days) as a complimentary therapy might have an impact on the incidence of illnesses. The aim of this study is to assess whether nutritional supplementation will significantly reduce both the incidence of malnutrition and the incidence of the three main childhood illnesses. If successful, this may prevent subsequent illness and break the vicious cycle of dependence between malnutrition and disease.

2. STUDY OBJECTIVES

The aim of the study is:

To determine the *effectiveness* of 14 days nutritional supplementation (RUTF or MNP) given concurrently with the appropriate medical treatment, amongst children diagnosed with malaria and/or diarrhoea and/or LRTI in reducing the incidence of acute malnutrition and the incidence of the 3 study diseases compared to a control group."

The primary objective is to determine the effectiveness of 14 days of RUTF supplementation versus no supplementation (control group) on the *incidence of acute malnutrition*
Primary Hypothesis: Supplementation for 14 days with RUTF, concurrently with appropriate medical treatment for malaria, and/or LRTI, and/or diarrhoea reduces the incidence of acute malnutrition compared to a control group during 6 months follow-up, amongst children 6-59 months of age with malaria and/or, LRTI and/or diarrhoea at time of recruitment.

Secondary objectives include:
Incidence of malnutrition
i.) Determine the efficacy of 14 days MNP supplementation versus a control group and RUTF versus MNP on the incidence of acute malnutrition during 6 months follow-up,

Frequency of morbidity
ii.) Determine the effect of 14 days supplementation on the *frequency of morbidity* (3 study diseases) during 6 months follow-up, comparing both intervention groups to the control group and comparing RUTF to MNP)

Weight changes immediately after intervention period, measured at day-14 and day-28
iii.) Measure *mean weight change* (total change and rate (grams/kg/day) of change), comparing both intervention groups separately (RUTF and MNP) to the control group
iv.) Determine *frequency of 'no weight gain and/or weight loss'*, comparing both intervention groups separately (RUTF and MNP) to the control group
v.) Assess if provision of 14 days of RUTF or MNP supplement promotes a sustained weight gain, measured after 28-days of follow-up, comparing both intervention groups separately (RUTF and MNP) to the control group

Sub group analyses
vi.) Moderately acutely malnourished at study inclusion: Determine the effect of 14 days supplementation on the *incidence of acute malnutrition* and *frequency of morbidity* (3 study diseases) during 6-months follow-up, comparing those moderately acutely malnourished at entry to those not acutely malnourished at entry.
vii.) Children under 3 years of age at inclusion: Determine the effect of 14 days supplementation on the *incidence of acute malnutrition* and *frequency of morbidity* (3 study diseases) during 6-months follow-up, comparing children under 3 years of age to older children.
viii.) Breastfed children: Determine the effect of 14 days supplementation on the *incidence of acute malnutrition* and mean weight gain (incl. rate) during 6-months follow-up, comparing breastfed children to those not breastfed.

3. STUDY DESIGN

This is a partially blinded randomized controlled trial with three study groups. Children 6 to 59 months of age presenting at the OPD in Goronyo and diagnosed with one or more of the three study diseases (malaria, diarrhoea and LRTI) are eligible for the study.

Children participating in this study will be randomised to one of 3 study groups to:

A) Receive 14 days of RUTF supplementation with standard care and treatment or

B) Receive 14 days MNP supplementation with standard care and treatment or

C) Be included in a control group receiving standard care and treatment but not receiving nutritional supplementation (control group)

Individual follow-up will be 6 months. During this time, children in the RUTF or MNP group will receive 14 days nutritional supplement every time diagnosed with at least one of the three study diseases, not exceeding more than 14 days supplementation in any 28 day period.

4. STUDY SITE

The study site is an MoH/MSF OPD located at the Mother and Child Health (MCH) Clinic in Goronyo, Goronyo LGA, Sokoto State, North West Nigeria. Médecins Sans Frontières, in collaboration with the Sokoto State Ministry of Health, supports the Mother Child Health (MCH) Clinic and operates a nutritional feeding programme for severely acutely malnourished children with both an inpatient stabilisation centre and an ambulatory therapeutic feeding program. The MCH clinic in Goronyo also includes an an inpatient department for children requiring hospitalisation. MSF support to the MoH includes provision of drugs, nutritional products and medical materials, clinical staff for supervision and direct patient care, supplementation to the salaries of MoH staff, and health care waste management,. All care is offered free of charge. Patients requiring more advanced secondary care are transferred to Sokoto city by MSF with all charges paid by MSF.

4.1 Number of Participants

The total study population will consist of 2,136 participants, 712 per study group. All children will be recruited at one study clinic site. The study team is expected to recruit approximately 15 participants per day, requiring approximately 143 working days (approximately 29 weeks based on a 5-day work week) for recruitment.

4.2 Inclusion criteria

Children will be included if they meet all the following inclusion criteria:

- 6 to 59 months of age
- Not malnourished or moderately acutely malnourished children
- Diagnosis of malaria and/or diarrhoea and/or LRTI
- Intending to remain in area for the duration of the 6 month follow-up
- Living within approximately 60 minutes walking distance from the clinic
- Informed consent from a guardian*

* Parent or other legal guardian. If not a parent, must be at least 18 years of age

4.3 Exclusion criteria

Children will not be included if they meet any one of the following exclusion criteria:

- Child is exclusively breastfeeding
- Child is severely malnourished
- Presence of ‘General Danger Signs’
- Presence of severe disease (including severe malaria, severe LRTI, severe diarrhoea)
- Needing hospitalisation for any reason
- Known history of allergy to the nutritional supplementation
- Having a sibling enrolled in the study*

*When one twin child is recruited, the twin sibling cannot be recruited but will receive the same supplement. This is done to limit the risk that, given the unique nature of twins, a guardian may share supplementation with twin siblings.

Patients who do not meet all eligibility requirements and/or who fulfil any one exclusion criteria at screening will be considered not eligible for participation (See Annex f1-screening form).

5. PARTICIPANT SELECTION AND ENROLMENT

5.1 Identifying participants

Study patients will be recruited from amongst routine users of the Goronyo OPD services. Initial diagnosis of the study diseases will be conducted at the Goronyo OPD by MoH clinical staff using MoH/MSF clinical diagnostic tools and protocols.

Patients diagnosed with one of the 3 study diseases at the OPD will receive drug prescriptions as needed, a brief explanation about the study, and be voluntarily referred to the study clinic for further screening for study inclusion.

5.2 Consenting participants

Guardians of the participants will be informed about the purpose of the study, the procedures involved when participating, and asked for their consent. Non parent guardians of the child must be older than 18 years of age to give consent.

Informed consent forms will be translated into local languages, back-translated and piloted for comprehension in the area of the study site. As a significant proportion of the population is not literate, the consent form will be read out to the patient's guardian to obtain oral consent as required (see annex f2-informed consent). Participants will receive a copy of the consent form for their reference.

It will be explained to the guardian that participation is voluntary and that patients will receive the same standard of medical treatment whether or not they agree to participate in the study. It will be further explained that patients can discontinue participation from the study at any time without explanation and without any negative impact on their future care and treatment.

5.3 Screening for eligibility

After referral to the study clinic, patients will be further informed about the study and screened by the study team.

A maximum daily number of children accepted for study screening will be defined according to the capacity of the study team. Based on information of how many OPD referrals the study team can expect per day, children referred will be selected systematically for further screening. As an example, if the study team can screen at most 30 children per day and they expect that 60 children will be referred to the study clinic from the OPD, every 2nd child will be screened for study inclusion. Selection for screening will be in the order of presentation to study clinic after referral from the OPD.

If the child is deemed eligible for the study according to inclusion and exclusion criteria, the guardian will be asked to voluntarily participate.

5.4 Ineligible and non-recruited participants

With limited screening capacity and a high volume of OPD patients with at least one of the study diseases, some children referred from the OPD to the study clinic will not be screened. If a child is not to be screened, the guardian will be informed of this immediately and be referred back to the MoH OPD to attain follow-up care or medications as per normal OPD protocol. This child will receive no further services from the study clinic.

6. RANDOMISATION

6.1 Randomisation

Simple randomisation will be implemented. There will be no reference to assigned study groups (e.g. no use of terminology such as group A, B, or C). Rather, the total patient numbers will be randomly allocated to an equal proportion of one of the three study groups.

The randomization list will be computer generated by a statistician.

6.2 Blinding

Owing to the nature of the product packaging and use of a control group without placebo, study participants will not be blinded to which study group they were assigned. It will also not be possible to blind staff distributing the supplement.

Study staff blinded to the allocation treatment includes:
- Principle investigator
- Co-investigators
- Field Research Coordinator
- All clinical and ancillary medical staff
- Technical staff collecting anthropometric measurements
- Questionnaire interviewers
- Data managers
- Statisticians

Every effort will be made to ensure that study staff remains blinded to the treatment allocation. Efforts will be made to inform participants and staff to avoid discussion that might reveal allocation.

The allocation scheme will be formally revealed only after the data from the last participant has been captured.

Each participant will be assigned a unique study identification number, used to identify the study participant in all procedures. Study numbers will be assigned sequentially as subjects enter the study. Once a number has been assigned, that number will not be used again (e.g. if a subject discontinues or a number is allocated incorrectly).

6.3 Treatment allocation

The study dispenser will have access to a register with all unique study numbers and the assigned intervention.
The study dispenser will allocate the study group to the participant when the participant collects the nutritional supplement. As such, the dispenser will be the only person of the study team to know the allocation scheme. The allocation register will be kept by the dispenser in such a manner that guarantees no unauthorized staff can have access. (see annex f9-dispensary allocation register)

7. NUTRITIONAL SUPPLEMENTATION

Guardians will be instructed in proper storage and consumption of the allocated nutritional supplement products. Refer to Annex 11 for the composition of these products.

7.1 RUTF (Plumpynut®)

Participants allocated to receive Plumpynut® will receive 14 sachets, one for each day.
One package of Plumpynut® provides 500 kcal and vitamins and minerals, including vitamin A, B, C, selenium, zinc, folic acid, copper, iron. Ingredients used for the manufacturing of Plumpynut® include peanuts and milk components. (see annex 1)

7.2 MNP (MixMe®)

The MNP contains the micronutrients recommended by WHO, UNICEF, and the WFP (see Annex 11).[25] In order to be approximately comparable with the micronutrient composition of RUTF the daily quantity is doubled. Participants allocated to receive MixMe® will receive 28 sachets, two sachets for each day of supplementation.

7.3 Control group

For the purpose of this study, it is not practically feasible to design and manufacture a placebo for RUTF or the MNP. Children randomly assigned to the control group will receive equivalent care and follow-up as the children assigned to a study group receiving supplementation. The only difference is

that they will not receive a nutritional supplement (current clinical practice does not include nutritional supplement).

8. CLINICAL PROCEDURES AND METHODOLOGY

8.1 Study clinic procedures at day of inclusion (day-0)

After inclusion, study procedures on day-0 include:

- Baseline socio-demographic questionnaire with i) Socio-economic data (incl. education level of guardian, whether family is receiving food aid, average number of meals consumed prior to clinical visit, etc.) and ii) Demographics (name, age, address) (see annex f4-General questionnaire)
- Child health questionnaire, including breastfeeding practices, duration of illness prior to clinical visit
- Anthropometric measurements, including weight, height, and MUAC
- Medical examination, including fever, concomitant illnesses, bilateral oedema
- Laboratory exams: haemoglobin, if indicated
- Counselling on the importance of providing their child with an extra meal during the convalescence period

Treatment of study diseases: All children will receive standard care and treatment according to current MSF and MoH medical protocols. This includes provision of zinc supplement to children with diarrhoea according to WHO and MSF recommendations.

DOT: For any drugs prescribed by the MoH OPD clinician, participants will be responsible for retrieving these drugs from the MoH pharmacy, as per normal practices. The first dose of prescribed drugs will be taken in the presence of a study nurse.

In addition to the three study diseases, participants presenting to the study clinic for follow-up visits (scheduled and unscheduled) will be cared for by the study clinic for any illnesses not requiring lengthy or complicated follow-up. Children suspected to have a serious illness will be referred to the MoH OPD or other appropriate services.

8.2 Follow-up schedule

After inclusion, the guardian will be instructed to return to the study clinic whenever the child is ill unless an emergency dictates attendance at a closer medical facility. Study staff will visit the OPD waiting room for study participants that might be mistakenly waiting there instead of going to the study clinic. MoH OPD will be asked to immediately refer all study participants to the adjacent study clinic.

In order to record any diagnoses and treatments prescribed when visiting a non-study clinic (i.e. private clinic, traditional healer, etc), participants be given a ‘study card’ describing the nature of the study and the need for non-study clinical staff to record on the card any new clinical data and medications provided or prescribed. Participants will be asked to actively provide this study card whenever consulting a non-study clinic. The decision to record diagnoses from a non-study clinic for study purposes will be made on individual basis, considering reliability and relevance of diagnosis (See annex f6 for the Patient Take-Home Card).

A number of procedures will be repeated during the follow-up visits at the study clinic. Patients will have a follow-up form for procedures to be ticked off when completed (see annex f3 - Study Follow-up Form). Table 1 below gives an overview over all study procedures.

Table 1: Schedule of study procedures

Procedure	Day 0	Day 14	Day 28 and every 4 weeks after	Unscheduled visits	Final visit (day 168)
Informed consent	√	-	-	-	-
Randomisation	√	-	-	-	-
Baseline questionnaire	√	-	-	-	-
Child health, history	√	√	√	√	√
Clinical examination*	√	√	√	√	√
Follow-up questionnaire	-	√	√	-	√
Weight	√	√	√	-	√

MUAC	√	√	√	-	√
Height	√		√	-	√
DOT	√	-	-	-	-
RUTF / MNP distributed	√	√§	√§	√§	
Compensation distributed	√	√	√	-	√

* Complete exam when diagnosed with an illness or otherwise deemed necessary, otherwise limited to a rapid exam, including assessing treatment side-effects and response
§ *When indicated*

Questionnaires: Questionnaires will be translated into local languages, back-translated and piloted for comprehension in the area of the study site. (See annex f4 for baseline questionnaire and annex f5 for follow-up questionnaire)

Unscheduled visits: If unscheduled visit occurs within 48 hours prior to a scheduled visit, the child will not need to return for that upcoming scheduled visit.

Repeated supplementation: Patients presenting with a new episode of a study disease will receive a new 14-day supplement, according to the intervention originally assigned. Though care and treatment will be available whenever ill, a child will not receive more than 14 days supplementation within any 28 day period. For example, a child receiving a new supplementation package on day-42 due to a newly diagnosed study disease cannot receive another supplementation package before day-70.

One reason to limit the distribution to a monthly frequency is to minimize potential bias related to the likelihood that children receiving Plumpynut® (a product known and recognized by the community) may be more likely to come forward for an unscheduled visit with the intention to receive additional distribution of supplementation.

8.3 Measuring malnutrition

Weight, height, MUAC and bilateral oedema will be measured upon inclusion and every following visit (scheduled and unscheduled). The nutritional status will be assessed using the weight-for-Height index according the WHO (2005) growth references, MUAC, and the presence of bilateral oedema.

8.3.1 Weight

Time of day: Because the weight of an individual varies during the day, weight will be measured at the same time of day on each follow up visit. With patients being recruited throughout the day, enrolment in the study and the first weight measured will be at any time on day-0.

To limit intra-day variation in weight, efforts will be made to ensure follow-up visits are scheduled at the same time of the day throughout the 6 month study follow-up. A weighing within 3 hours on either side of the original weighing will be accepted. If a patient arrives outside this limit, the participant will be asked to return the next day within the proper time window.

Weighing procedures: Two weighing scales, both SECA model 835, a very precise electronic scale, will be used for small babies and toddlers sitting or standing on the scale. Below weights of 20 kg the scale has a precision of +/-20 grams and for heavier weights up to 50 kg a precision of +/-50 grams. To minimize inaccuracy due to a child's movement, great care will be taken to place the scale level and on a stable surface and every effort will be made to ensure that restless children are kept calm during the weighing procedure. Quality control measures will be taken to ensure scale accuracy and the scales will be checked daily by a constant weight of 10Kg.

The weighing will be done by the same team of two persons throughout the study, monitored and supervised closely by the field research coordinator (FRC). All children will be weighed undressed and without jewellery or other ornaments. One person will perform the weighing (ensuring scale is correctly positioned, correctly calibrated, and assisting the guardian with placing the child on the scale). The weight will be taken when the child is relatively still. This same person will read out loud the weight measured. The second person will visually observe the weight on the digital scale to confirm that it has been correctly read out loud and the second person will verbally repeat the weight and record the confirmed result on the appropriate form.

8.3.2 Height

In order to detect even small increases in the height of a child, height will be measured at day-0 and every monthly visit to the clinic. Two persons will use a precision height board (infant-child-adult measuring board, aluminium, Promes). One person will read out loud the height measured and the second person will verbally repeat the height for confirmation and record the result on the appropriate form.

8.3.3 MUAC

MUAC will be measured using an standard MUAC tape at day 0 and at the monthly visits. One person will read out loud the MUAC measurement and the second person will verbally repeat the measurement and record the result on the appropriate form.

8.4 Three study diseases

Three diseases/morbidities are targeted during this study: malaria, diarrhoea, and LRTI. Clinical diagnoses will be made using existing standard MSF/WHO protocols and case definitions [26, 27] A flow chart with to guide diagnosis and inclusion is detailed in Annex f7-diagnostics.doc.

- Diarrhoea:

The forms of diarrhoea include:

1. Acute diarrhoea, simple without blood: the passage of at least three liquid stools per day for less than 2 weeks
2. Persistent/chronic diarrhoea: diarrhoea beginning at least 14 days ago, and any period without diarrhoea has not exceeded two days. (WHO definition) [28]
3. Bloody diarrhoea

- Lower Respiratory Tract Infection (LRTI)
  Refers to acute pneumonia:
  - children presenting with cough or difficulty breathing **plus**
  - Increased respiratory rate
    - Age 6–11 months: RR > 50 breaths /minute
    - Age 12 to 59 months: RR > 40 breaths /minute

- Malaria, Plasmodium falciparum
    - Fever or history of fever within 24 hours with positive malaria RDT result

8.5 Compliance

Compliance regarding the consumption and acceptability of the nutritional supplements will be assessed only after the first 14 days of supplementation, through a questionnaire and collection of returned supplement sachets (used or unopened). The study dispenser will count and record the returned sachets (empty or unopened) after the questionnaire is completed.

8.6 Emergency unblinding procedures

Only when a serious adverse event occurs and it is deemed necessary to unblind a patient's intervention group will that patient's allocation be revealed.

8.7 Withdrawal procedures

Children will be withdrawn from the study if:

i.) Participant fulfils criteria for severe malnutrition during follow-up period Participant who is moderately malnourished at inclusion experiences rapid weight loss (≥10% in 28 days)
ii.) There is a serious protocol violation
iii.) Participant is erroneously included
iv.) There is the risk that the nutritional supplement would negatively influence a prescribed medical protocol (i.e. medical treatment has priority).

Patients who become severely malnourished and/or patients moderately acutely malnourished at inclusion who experience rapid weight loss (loss of ≥10% in 28 days) will be referred to the existing MSF Therapeutic Feeding Program for care and treatment.

Participants who are withdrawn from the study will continue to be followed up if possible and monitored following standard study protocol. The use of data for analysis from withdrawn participants will be assessed by the study statistician.

8.8 Endpoints

8.8.1 Primary endpoint

The primary endpoint is "negative nutritional outcome (NNO)" of a child within the 6 months follow-up period. The incidence of a negative nutritional outcome will be defined in two different ways according to the baseline nutritional status.

i) for children with no malnourishment at time of entry into study, "negative nutritional outcome" is defined as progression to moderate or severe malnourishment
ii) for children with moderate malnourishment at time of entry into study, "negative nutritional outcome" is defined as loss of ≥10% of baseline weight or progression to severe malnourishment, whichever is reached first.

Thus, the event of a child reaching a "negative nutritional outcome" at least once within 6 months of follow-up period will be compared between the three study groups.

8.8.2 Secondary endpoints

Secondary endpoints include

- Number of NNO's per study group in a period of 6 months
- Time to NNO
- Number of new events of a study disease in 6 months
- Weight change at 14 days and 28 days after study inclusion, and at end of study period
- Rate of weight change (grams/kg/day) at 14 days and 28 days after study inclusion, and at end of study period
- Proportion of children not gaining weight (including losing weight), measured at day-14 and day-28 after study inclusion.
- Proportion of children developing bilateral oedema
- Proportion of moderate acutely malnourished children improving to a non acutely malnourished state
- Mortality: overall mortality,
- Morbidity, including the three study diseases
- Proportion of children with a reported cough at clinical visit

8.9 Data collection

An overview of forms where data are recorded is given in annex f10-overview data flow. Data will be routinely captured using EpiData software program, including double data entry to ensure quality data capturing.

Data will be routinely sent to the MSF Study PI and epidemiologist based in Amsterdam for routine quality control. To minimise missing data during the trial, the database will be checked routinely. Any critical data missing will be obtained by questioning staff and, if necessary, the guardian.

In an effort to minimise missing data, participants will be reminded of their scheduled visit 24 hours prior to the scheduled visit by means of mobile telephone. If a patient misses an appointment, the participant will be traced by outreach workers to ensure follow-up. When possible, information on the reason for missing data will be recorded.

Study data will be kept on the study site and handled confidentially. After study completion, all records will be stored in an appropriate manner at MSF offices at capital level for long-term storage. All data (paper records and electronic data) will remain the property of MSF-OCA, who will commit to retain the data for 5 years.

MoH OPD patient medical cards will remain the property of the patient and/or health facility, as per

current standard procedures in the routine health services.

9. DATA ANALYSIS

9.1 Sample size

The sample size is based on the primary hypothesis: Supplementation for 14 days with RUTF, concurrent to appropriate medical treatment for malaria, and/or LRTI, and/or diarrhoea reduces the incidence of malnutrition.

The sample size calculation is based on a combined incidence of moderate and severe malnutrition amongst children who are sick.

There are hardly studies reporting the incidence of malnutrition and no studies reporting incidence of malnutrition among sick children. Therefore for the purpose of these calculations, we assume that the incidence is estimated by dividing the prevalence of malnutrition by the period of having malnutrition times 6 months: = [(Prevalence / Duration of moderate malnutrition) * 6 months]
The estimated incidence for moderate and for severe malnutrition will be added to result in the incidence of negative outcome as defined as primary endpoint of this study.

The prevalence of moderate malnutrition among sick children is 15.5% and the prevalence of severely malnutrition among sick children is 7.2% according to a survey at February 2009 at start of the program.

Literature gives a duration of malnutrition (untreated) is estimated to be 7.5 months [29] and the duration of severe malnutrition is estimated at 4 months at the time of the survey. As duration of malnutrition might be different in the context of Goronyo we varied assumptions in order to use the lowest incidence as a basis for the sample size. This results in sample size that is large enough to give enough statistical power for the primary hypothesis (the sample size increases with a decreasing incidence). Increasing the prevalence of malnutrition in general or decreasing the duration of severe malnutrition (as there is treatment available) resulted in a higher incidence, but prolonging the duration of moderate malnutrition to 10 months gives a lower incidence.

Therefore the incidence is based on duration of 4 months for severe malnutrition and duration of 10 months for moderate malnutrition.

This results in an incidence of 0.201 per 6 months

Formula: 0.201 = [(0.155 / 10 + 0.072 / 4)] * 6

The assumed incidence rate in the control group (baseline incidence) is 0.20. A 30% reduction in the supplementation group, which is considered a clinically and operationally relevant improvement, results in an assumed incidence rate of 0.14 in each of the two interventions groups.

A Poisson regression with a binary independent group variable with proportion of 0.5 (equal allocation ratio) using a sample of 1320 observations (i.e. 660 per group) achieves 80% power at a 0.05 significance level to detect a incidence rate ratio of at least 0.7 (i.e. incidence rates of 0.14 (intervention rate) vs. 0.2 (baseline rate)).

To account for an assumed drop-out rate of approximately 10%, 734 children will be included in each group (i.e. 2202 in total for all three groups).

The sample size estimation is based on the first two hierarchical test hypotheses (while the third hypothesis comparing RUTF with MNP for non-inferiority is considered explorative). Sample size estimations are performed with PASS 2008 (Version 08.0.13, Windows XP) and SAS version 9.2 (SAS Institute, Cary, NC, USA).

9.2 Populations for analysis

<u>Intention to treat population</u>

The intention to treat population (ITT) includes all patients randomised that received at least one dose of study medication and who have baseline data recorded.

Per protocol Population

The per protocol population (PP) includes all patients of the ITT population which were treated following the specifications of the study protocol without major protocol deviations. Excluded from the per protocol population will be patients that meet at least one of the following criteria:

- Violation of one of the following exclusion criteria: exclusive breastfeeding, hospitalisation for serious complicated illness (e.g. chronic illness).
- Not treated according to the allocated intervention group
- Patient misses more than 2 monthly scheduled visits
- Patient misses 2 or more consecutive monthly scheduled visits
- Participation in other feeding programmes

Completed the study

Patients will be considered as having 'completed the study' if they have attended at least the first and last scheduled visit at 6 months.

Baseline drop-out population

All patients enrolled who dropped out before the start of randomized treatment.

9.3 Descriptive Analyses

All available data will be analysed descriptively for each intervention group. Results will be summarised as frequencies and percentages for nominal data; as means, standard deviations, range, and 95%-confidence intervals for continuous data; and as medians, quartiles, and ranges for ordinal data.
Differences regarding baseline variables will be analysed by appropriate tests (e.g. Chi squared test, Kruskal-Wallis test, or analyses of variance) in pair wise comparisons between the three treatment groups, and will additionally be checked for clinical relevance.

9.3.1 Primary analysis of the primary endpoint

The primary endpoint is "negative nutritional outcome" of a child within the 6 months follow-up period as defined in section 8.8.1. The incidence rate (in formulae below referred to simply as 'rate') of "negative nutritional outcome" will be computed as the number of events divided by total observed person-time in each treatment group. As an "event" the first time a child reaches a negative nutritional outcome will be used. The primary analysis of the primary endpoint will be performed on the intention to treat population with no imputation of missing data.

In the primary analysis, RUTF will be compared to the control group
The hypotheses in this step are

H_0: $rate_{RUTF} = rate_{Control}$
vs.
H1: $rate_{RUTF} \neq rate_{Control}$

where $rate_{RUTF}$= rate of "negative nutritional outcome" in the children in the RUTF group, and $rate_{Control}$= rate of "negative nutritional outcome" in the children in the control group.

The comparison of rates among the treatment groups will be performed by a Poisson regression model, adjusted for nutritional status at enrolment (fixed effect) and will be performed within one model including the three intervention groups with contrasts for each two-group comparison. Tests will be two-sided with a significance level of 0.05.

9.3.2 Secondary analysis of the primary endpoint

Although the study is primarily powered for the comparison between the RUTF and control group the other groups will be compared as well, keeping in mind that the sample size might not be large enough to obtain significant results.
Since there are three interventional groups to be compared, a hierarchical test procedure will be used to account for multiplicity.

1) In a first step, RUTF will be compared to the control group which is described above as analysis of primary outcome
If the RUTF group is not significantly different at a 0.05 level (two-sided test), the hierarchical procedure is stopped (all following analyses will be considered explorative). If the RUTF group is significantly different at a 0.05 level, the hierarchical procedure is continued with step 2.

2) In the second step, MNP will be compared to the control group
The hypotheses in this step are

H_0: $rate_{MNP} = rate_{Control}$
vs.
H1: $rate_{MNP} \neq rate_{Control}$

where $rate_{MNP}$=rate of “negative nutritional outcome” in the children in the MNP group, and $rate_{Control}$=rate of “negative nutritional outcome” in the children in the control group.

If the treatment factor is not significant at a 0.05 level (two-sided test), the hierarchical procedure is stopped (all following analyses will be considered explorative). If the treatment factor is significant at a 0.05 level, the hierarchical procedure is continued with step 3.

3) In the third step, RUTF will be compared to MNP (non-inferiority test).
The hypotheses in this step are

H_0: $rate_{RUTF} - rate_{MNP} > -\delta$
vs.
H1: $rate_{RUTF} - rate_{MNP} \leq -\delta$

where $rate_{RUTF}$=rate of “negative nutritional outcome” in the children in the RUTF group, $rate_{MNP}$=rate of ““negative nutritional outcome” in the children in the MNP group, and δ=inferiority margin.

As non-inferiority margin, a δ of 2% will be used (i.e. allowing a slightly worse result in the MNP compared to the RUTF group). The third step will be tested at a 0.025 level (one-sided test), by a one-sided 97.5% confidence interval of the difference in rates between the RUTF and the MNP group.

In addition, in case of significant and/or relevant differences of baseline values additional adjustment(s) for these factors will be performed within the model described above (in models with and without interaction), to address the potentially confounding or effect modifying effect of other variables. These variables include age (in months and binary: <36 months, ≥36 months), gender, breastfeeding practices, cough, underlying illnesses, socio-economic factors, food availability, length of illness before visiting MSF clinic, other health support sought during study, season during which child was in the study.

The amount and pattern of missing data of the primary endpoint will be checked by a person blinded to treatment allocation, and a decision based upon the result of this check will be made as to whether and how the imputation of missing primary endpoint data will be performed.

Additionally, the 6-months follow-up period will be split and analysed separately for 1 month periods with the method described above.

The analysis of the primary endpoints will also be repeated as described above separately for children with no malnourishment at time of entry into study and children with moderate malnourishment at time of entry into study.

The analysis of the primary endpoint will be repeated as described above for the per protocol population.

9.3.3 Analyses of secondary endpoints

All secondary endpoints (as defined in section 8.8.2) will be compared between the three treatment groups as described above (p-values will be considered explorative). In addition to the Poisson regression model, logistic regression model, analysis of variance (ANOVA), analysis of covariance (ANCOVA) and non-parametric analysis of variance will be used, according to the type of endpoint.

9.3.4 Pooled analysis

Prior to this study a similar study (pilot) had been conducted in Katanga, DRC, and a similar study will be conducted in Karamoja, Uganda. After finalizing the studies the data from the three studies will be pooled and analysed jointly with adjustment for the study location.

9.3.5 Statistical analysis plan and software

A detailed statistical analysis plan (SAP) will be developed prior to the data analysis. Software used will be SAS for Windows, Version 9.2 or higher (SAS Institute, Cary, NC, USA) and STATA, Version 11 (StataCorp, College Station, Texas, USA).

10. QUALITY CONTROL

10.1 Coordination

A field research coordinator will be dedicated to ongoing supervision and monitoring of study implementation. Clinical procedures and data collection will be evaluated regularly by the FRC. Other QC measures will include daily review of patient records, observation of interviews and clinical procedures, and ongoing evaluation of malaria laboratory procedures according to standardised checklists.

The PI and OCA epidemiologist will be present for the set up of the study site prior to implementation. They will also be available to conduct site visits every 3 months and make additional support visits as needed.

10.2 Diagnostics

Malaria RDT: Routine quality control procedures will include close monitoring and supervision of RDT performance against standardized monitoring checklists and blood films collected on a monthly basis including 15 positives and 15 negative samples to be crosschecked by expert microscopy as per the MSF OCA quality control protocol.

10.3 Staff, Training, and Supervision

In addition to the FRC, the study will have a dedicated team, including medical doctor, nurses, study assistants (incl. collection of weight and height measurements, participant tracers, supplement dispenser, receptionist). Every effort will be made to identify and recruit Nigerian staff, including the FRC.

Prior to study implementation, the PI and OCA epidemiologist, in collaboration with the FRC, will provide initial training and support in study methodology and implementation. Targeted training will be provided for specific team roles and responsibilities, including clinical diagnostic algorithms, proper use of the malaria RDT, technique in conducting objective questionnaire interviews, and accurate weight and height measurements.
In addition, staff will be briefed on the MSF ways of working, its role and mandate.

11. ETHICAL CONSIDERATIONS

The study will be performed in accordance with ethical principles that have their origin in the Declaration of Helsinki and are consistent with International Conference on Harmonisation (ICH)/Good Clinical Practice (GCP) and all applicable regulatory requirements.

11.1 Treatment of participants

Confidentiality

The medical examinations and the interviews will be performed in a private setting to ensure patient

confidentiality. Patient data will only be accessible to the relevant clinical staff and not accessible to any unauthorized person. In further data processing, patient data will be pseudonymous, and identified only by the study number.

Optimal medical treatment
Patient management will be based at all times on their clinical condition, independent of participation in the study. Participation in the study will not, at anytime, preclude supplementary treatment or a change in therapy if clinically indicated. The well-being of the patient will always be prioritised over study participation.
The study does not interfere with routine care normally delivered to children presenting to the Goronyo OPD. Children requiring hospitalisation on presentation will not be recruited into the study, nor will children who are severely acutely malnourished. Moderately malnourished children will be included in the study. This is because neither MSF nor the MoH provides specific nutritional intervention to children with MAM in Sokoto. This is due to the change-over to the new WHO standards (2006) which classify a larger group of children as severely malnourished. This results in many of the children previously defined as moderately malnourished, being included in programs for SAM.

Burden on study participants:
Individual study participants will be asked to attend for minimum 8 visits. A small compensation for time spent on travel and at the study clinic will be given after each scheduled clinical visit, e.g. a bar of soap. At the end of the study a larger compensation will be provided, e.g. a bednet and soap. The total costs of the compensation will be equivalent to the market value of the approximate average duration of travel and time spent in the clinic for the research. This will be approximately 10 hours.

Choices for the products will be made by the Field Study Coordinator in close collaboration with a diversity of stakeholders within the community (incl. mothers, community health workers, District Health Officer) and in observation of market prices.

11.2 Cooperation with national and local partners

Collaboration and cooperation between MSF-OCA and Nigerian health authorities (Goronyo LGA, Sokoto State and Federal Health Authorities) is critical for the success of this study and uptake of results if positive outcomes are demonstrated. It will be essential to ensure access to and provision of study information to key policy in combating malnutrition. Permission to run the study will be obtained from the State Ministry of Health. In addition, the local administrative authorities will be consulted about the nature and time-schedule of the planned study.

The details of the study and its implications will be discussed with all relevant stakeholders, at local and national levels.

Active collaboration with the main university paediatric hospital in Sokoto is ongoing through a paediatrician who forms part of the study team. He has provided input on the study protocol, and will present the study for local ethical review through the university. He will be an active member of the research team throughout the study and will be responsible for disseminating the research results through the paediatric and academic community in Sokoto state.

As the study involves a temporary intervention with a temporary benefit for those receiving an active intervention, it is essential that community members understand the purpose of the investigation and agree with the procedures. MSF-OCA field visitors will consult with village chiefs and community members, especially targeting women with young children throughout the study to ensure understanding and ongoing community acceptance of the study.

11.3 Benefits to the community

. If shown to be effective, this would contribute to reducing morbidity and mortality amongst the children in the community. Based on the positive outcome of this study, MSF will adapt protocols to include provision of nutritional supplementation for children with serious acute illness. Assuming positive benefit of the intervention, MSF will advocate with the MoH, and with donors for provision of the supplement. While it is accepted that RUTF is an expensive intervention and may be difficult for the MoH to implement alone, previous MSF prevention studies for malnutrition have resulted in uptake of the RUTF distribution strategy by UNICEF and WFP. If the study shows MNP to be effective, we anticipate this to be an intervention that can be implemented with minimal cost impact. In addition, we

would consider cost effectiveness studies if this was needed to convince decision-makers to implement the nutritional intervention.

The study also hopes to contribute to an improved understanding within the community, including medical staff, that good nutrition is essential after during and an acute infection.

Study participants will further benefit from monthly routine screening, intensified follow-up treatment and care.

Finally we want to address the issue of commercial interest. Plumpynut® is under patent protection currently. The Access to Medicines Campaign of MSF has been actively following this issue for the last years, and currently the patent is under pressure in a number of countries. More manufacturers are now producing RUTF, for example Compact produces a nutritionally equivalent product, called EeZeePaste® which is now used in some MSF programs. MixMe® is a product that as stated is widely used and similarly to another product on the market. It is distributed by UNICEF through a donation from the manufacturing company. We therefore do not see any direct commercial interest accruing as a result of this study to either the makers of Plumpynut® or MixMe®.

11.4 Feedback of results

Individual child results will be shared and discussed with the guardian in such a manner to not jeopardize established blinding of the study interventions. At the end of the study, feedback and preliminary outcomes will be provided to the community and to medical authorities.

11.5 Potential risks

The potential risk due to a nutritional supplementation is very low. The supplementation products have a history of proven effectiveness and benefit in similar settings. Nonetheless, any intervention carries with it a certain level of risk and, as such, participants must be monitored closely.

A paper by Caulfield *et al.* [30] reviewed the relation between iron supplementation and risk of reduced malaria parasite clearance rates and increased risk of treatment failure. However, these studies have been criticised on several points, including the use of a malaria treatment (sulfadoxine - pyrimethamine) in an area with parasite resistance to these drug components. [31]

Caulfield et al. conclude that no consensus exists on the risks of malaria morbidity and mortality associated with iron supplementation and that the alleviation of anaemia through iron supplementation is likely to have a far greater net benefit for an iron-deficient population, including those in malaria-endemic regions. The WHO concludes that routine iron supplementation is prioritised even in malaria endemic regions. [32] A recent Cochrane review (2009) that includes the studies mentioned above in their review, concludes that iron does not increase the risk of clinical malaria or death, when regular malaria surveillance and treatment services are provided.[33]

11.6 Adverse events

An **adverse event** (AE) is any untoward medical event affecting a clinical trial participant.

Each adverse event should be evaluated whether it is potentially directly related to the RUTF or MNP supplementation, e.g. vomiting, rash, etc. An **adverse reaction** (AR) is where it is suspected that an adverse event has been caused by a reaction to one of the supplements.

In this study the primary (incidence of malnutrition) and several secondary outcomes (e.g. incidence of disease) are per definition an adverse event but these will not be considered as an adverse reaction.

A serious adverse event /reaction

- results in death;
- is life threatening (i.e. the subject was at risk of death at the time of the event; it does not refer to an event which hypothetically might have caused death if it were more severe);
- requires hospitalisation; (excluding a therapeutic feeding centre)
- results in persistent or significant disability or incapacity;

Participants will be instructed to contact the clinic if any medical condition develops. The study team will examine the patient and will contact the FRC when a serious adverse event or a suspected adverse reaction (serious or not) occurs. The FRC will report the information to the PI within 24–48 hours of becoming aware of the event. The FRC will initiate the appropriate care and treatment according to their medical judgment.

The report includes study group, type of event, onset date, Investigator assessment of severity and causality, date of resolution as well as treatment required, investigations needed and outcome.(see annex F8-serious adverse event/reaction). Depending on severity, when an SAE / SAR occurs, the FRC will review all documentation (e.g. hospital notes, laboratory and diagnostic reports) related to the event. Alternative causes such as natural history of the underlying disease, concomitant therapy, other risk factors and the temporal relationship of the event to the supplementation will be considered.

A subgroup of the study advisory group (including the OCA Medical Director, the Primary Investigator, and the paediatrician of UDUTH Sokoto) will continuously review the occurrence of all serious adverse events and adverse reactions. On authority of the OCA Medical Director or MoH of Sokoto the study can be interrupted, altered or stopped as a result of the investigation of an SAE or SAR.

11.7 Interim analysis and Data and Safety Monitoring Board

An interim analysis is not required in this study. The study sample size and the 6-month follow-up period is the minimum needed before having the statistical power to show evidence of any significant (operational or statistical) difference in the primary outcome (negative nutritional outcome; 'NNO') when comparing study groups.

In addition, occurrence of SAE's, SAR's and mortality will be monitored, evaluated and reported routinely throughout the study. A subgroup of the study advisory group (including the OCA Medical Director, the Primary Investigator, and the paediatrician of UDUTH Sokoto) will continuously review the occurrence of all SAEs and SARs. On authority of the OCA Medical Director or the MoH of Sokoto the study can be interrupted, altered or stopped as a result of these investigations. The MSF ERB and local ERB/MoH will be immediately informed if this is the case.

Due to the proven safety of the supplementation products and because the products are not used in a manner very different than that already proven safe, the use of an independent Data and Safety Monitoring Board (DSMB) is not warranted. If the advisory committee is unable to make a decision on the outcome of the interim analysis, an independent body external to MSF will be immediately consulted.

12. STUDY INVESTIGATORS AND THEIR ROLES

The Public Health Department of MSF-OCA is responsible for the study. Only on the authority of the Medical Director of MSFOCA or the MoH of Nigeria should the study be substantially altered or stopped. The principal investigator (PI), S van der Kam, is accountable to MSF-OCA.
An advisory committee will advise the PI at crucial stages on design and implementation of the study and includes:

Name	Title	Role	Afiliation
Kam, van der S.	Principle Investigator	Responsible for the research including protocol development, implementation, analysis and report writing. Guarding the budget, represent the research internally and externally.	MSFOCA, Amsterdam, The Netherlands
Swarthout, T	Epidemiologist	Contributing to study design, implementation in the field and writing of protocols and reports; guiding data management, advice on interpretation of results.	MSFOCA

Boele van Hensbroek, M	Paediatrician	Providing advice on protocol development, specifically medical and paediatric issues, advice on interpretation of results	Global Child Health Group, EKZ-AMC Academic medical Centre, Amsterdam
Gómez Restrepo, C	Medical Coordinator, Nigeria	Providing advice on issues related to local circumstances and overall organization of implementation of the study and responsible for all communications with the authorities concerning the study.	MSFOCA
Not identified	Field Research Coordinator (FRC)	Responsible for hiring and training of staff, pilot protocol, organise implementation, ensuring the quality of diagnosis, treatment, procedures, the case reports, follow-up visits and data entry. Communicate directly with PI, Medco, epidemiologist when required.	MSFOCA
Jiya, N M	Paediatrician	Advice in study design, responsible for ethical approval process of the Nigerian ethical review board, represent study to the Nigerian government and population,. Liase with appropriate Nigerian research institute. Member of the safety committee to review any SAE. Input as member of study group to interpretation of results of study and formulation of recommendations and conclusions.	Usman Danfodio University Teaching Hospital (UDUTH), Sokoto, Nigeria
Roll, S	Statistician	Responsible for sampling frame, statistical procedures and data analysis Advice on study design, interpretation of results and formulation of recommendations	Institute for Social medicine, Epidemiology and Health Economics Charité University Medical Center, Berlin Germany
Tinnemann, P	Researcher	Advice in the research design and feasibility, implementation, interpretation of results and formulation of recommendation. Provide medical advice, guard scientific level and relevance	Institute for Social medicine, Epidemiology and Health Economics Charité University Medical Center, Berlin Germany
Shanks, L	OCA Medical Director	Advice in the research design and feasibility, critical review of research protocol, interpretation of results and formulation of recommendation. Responsible for ethical review procedures of MSF, approve budgets, external communication (protocol, report). Final responsible to review any SAE within the safety committee. Institutional hierarchical responsible	MSFOCA,

Authorship

The PI is responsible for drafting the report of this study, in collaboration with the OCA epidemiologist. Publication in an appropriate medical scientific journal will be sought. The PI is responsible for ensuring the collaboration and consent of all co-workers and for submitting a draft version of the final report to

the advisory committee within three months of completion of the study.

Authors will be drawn from the advisory committee and will be considered to be authors on 1) substantial contributions to conception and design, acquisition of data, or analysis and interpretation of data; 2) drafting the article or revising it critically for important intellectual content; and 3) final approval of the version to be published. Authors should meet conditions 1, 2, and 3.

Other members of the study group will be acknowledged as contributors in any publications or presentations.

13. REFERENCES

1. Black, R., et al., *Maternal and child undernutrition: global and regional exposures and health consequences.* The Lancet, 2008. **371**(9608): p. 243-260.
2. Goulet O, L.E., Branski D, Martin A, Antoine J, Jones PJH. , *Nutritional solutions to major health problems of preschool children: how to optimise growth en development.* J Pediatr Gastroenterol Nutr 2006(S1-S3).
3. Rowland, M., S. Rowland, and T. Cole, *Impact of infection on the growth of children from 0 to 2 years in an urban West African community*. 1988, Am Soc Nutrition. p. 134-138.
4. Rowland, M., T. Cole, and R. Whitehead, *A quantitative study into the role of infection in determining nutritional status in Gambian village children.* The British journal of nutrition, 1977. **37**: p. 441-441.
5. Friedman, J., et al., *Malaria is related to decreased nutritional status among male adolescents and adults in the setting of intense perennial transmission.* The Journal of infectious diseases, 2003. **188**(3): p. 449-457.
6. Nyakeriga, A., et al., *Malaria and nutritional status in children living on the coast of Kenya.* American Journal of Clinical Nutrition, 2004. **80**(6): p. 1604.
7. Shiff C, C.W., Winch P, Premij Z, Minjas J, Lubega P. , *Changes in weight gain and anaemia attributable to malaria in children living under holoendemic conditions. .* Trans Royal Soc Trop Med Hyg 1996. **90**: p. 4.
8. Tomkins, A., *Protein–energy malnutrition and risk of infection.* Proceedings of the Nutrition Society, 1986. **45**(03): p. 289-304.
9. Bahwere, P., et al., *Improvements in nutritional management as a determinant of reduced mortality from community-acquired lower respiratory tract infection in hospitalized children from rural central Africa.* Pediatric Infectious Disease Journal, 2004. **23**(8): p. 739-747.
10. Hoare, S., et al., *Dietary supplementation and rapid catch-up growth after acute diarrhoea in childhood.* The British journal of nutrition, 1996. **76**(4): p. 479-490.
11. WHO, *Food, water and health, a manual for community educators.* 1994.
12. Ramakrishnan, U., et al., *Multi-micronutrient supplements but not vitamin A or iron supplements alone improve child growth: results of three meta-analyses.* J Nutr, 2004. **134**: p. 2592-2602.
13. Aggarwal, R., J. Sentz, and M. Miller, *Role of zinc administration in prevention of childhood diarrhea and respiratory illnesses: a meta-analysis.* Pediatrics, 2007. **119**(6): p. 1120.
14. Miller, E., et al., *Meta-analysis: high-dosage vitamin E supplementation may increase all-cause mortality.* Annals of Internal Medicine, 2005. **142**(1): p. 37.
15. Kam, v.d.S.S., T; Niragira; O. Froud, A; Mukonema Sompwe, E;Mills, C; Shanks,L; Roll, S; Tinnemann, P., *The effectiveness of ready-to-use therapeutic food (RUTF) for catch-up growth after an episode of P.falciparum malaria.* planned, 2010.
16. Defourny, I., et al., *Management of moderate acute malnutrition with RUTF in Niger.* Field Exchange 2007. **31**.
17. Diop, E., et al., *Comparison of the efficacy of a solid ready-to-use food and a liquid, milk-based diet for the rehabilitation of severely malnourished children: a randomized trial.* American Journal of Clinical Nutrition, 2003. **78**(2): p. 302.
18. Linneman, Z., et al., *A large-scale operational study of home-based therapy with ready-to-use therapeutic food in childhood malnutrition in Malawi.* Maternal and Child Nutrition, 2007. **3**(3): p. 206-215.

19. Isanaka, S., et al., *Effect of preventive supplementation with ready-to-use therapeutic food on the nutritional status, mortality, and morbidity of children aged 6 to 60 months in Niger: a cluster randomized trial.* JAMA, 2009. **301**(3): p. 277.
20. WHO , W., UNICEF, *Preventing and controling micronutrient deficiencies in populations affected by an emergency. Multiple vitamin and mineral supplements for pregnant and lactating women, and for children aged 6 to 59 months. Joint statement by the World Health Organisation, World Food Program and the United Nation's Children Fund*. 2007.
21. WFP, D., UNHCR, *Micronutrient Powder (MixMe™); Program for Under-fives and Pregnant and Lactating Women Affected by Cyclone Sidr in Bangladesh (Asia)* 2009.
22. Zlotkin, S., et al., *Micronutrient sprinkles to control childhood anaemia.* PLoS Medicine, 2005. **2**(1): p. e1.
23. Zlotkin, S., et al., *Micronutrient sprinkles to control childhood anaemia.* PLoS Med, 2005. **2**(1): p. e1.
24. WFP, D., UNHCR, *Micronutrient Powder Use and Outcomes in Refugee Camps in Nepal (Asia)*, in *Implementing, improving nutrition, improving lives*. 2009.
25. WHO, W., UNICEF, *Preventing and controling micronutrient deficiencies in populations affected by an emergency. Multiple vitamin and mineral supplements for pregnant and lactating women, and for children aged 6 to 59 months. Joint statement by the World Health Organisation, World Food Program and the United Nation's Children Fund*. 2007.
26. WHO, *Pocket book of hospital care for children; guidelines for the management of common illnesses with limited resources*. 2005.
27. MSF, *Clinical guidelines, diagnosis and treatment manual, 8th edition*. 2009.
28. WHO, *The treatment of diarrhoea; a manual for physicians and other senior health workers* 2005.
29. Garenne, M., et al., *Incidence and duration of severe wasting in two African populations.* Public Health Nutrition, 2009: p. 1-9.
30. Caulfield, L., S. Richard, and R. Black, *Undernutrition as an underlying cause of malaria morbidity and mortality in children less than five years old.* The American journal of tropical medicine and hygiene, 2004. **71**(90020): p. 55-63.
31. English, M. and R. Snow, *Iron and folic acid supplementation and malaria risk.* The Lancet, 2006. **367**(9505): p. 90-91.
32. WHO, *Iron supplementation of young children in regions where malaria transmission is intense and infectious disease highly prevalent*, in *Statement*. 2006.
33. Ojukwu, J., et al., *Oral iron supplementation for preventing or treating anaemia among children in malaria-endemic areas.* status and date: Edited (no change to conclusions), published in, 2009. **4**.
