# Supplementary material for: Effect of Short-Term Supplementation with Ready-to-Use Therapeutic Food or Micronutrients for Children after Illness for Prevention of Malnutrition: A Randomised Controlled Trial in Nigeria
Source: PLoS Med. 2016 Feb 9;13(2):e1001952. doi: 10.1371/journal.pmed.1001952 (PMC4747530; doi:10.1371/journal.pmed.1001952)
Supplement: S3 Text — (PDF) [file pmed.1001952.s003.pdf]

CONSORT Checklist

Supplement to:

Effect of Short-Term Supplementation with Ready-To-Use Therapeutic Food or Micronutrients for Children after Illness on Prevention of Malnutrition: A Randomised Controlled Trial in Nigeria

Saskia van der Kam[1,2*], Nuria Salse-Ubach[3], Stephanie Roll[4], Todd Swarthout[1], Sayaka Gayton-Toyoshima[1], Nma Mohammed Jiya[5], Akiko Matsumoto[1], Leslie Shanks[1]

[1]Médecins Sans Frontières (MSF), Amsterdam, Netherlands; [2]Ecole de Santé Publique, Centre de Recherche en Politiques et Systèmes de Santé-Santé Internationale Université Libre de Bruxelles, Brussels, Belgium; [3]MSF, Barcelona, Spain; [4]Institute for Social Medicine, Epidemiology and Health Economics, Charité- Universitätsmedizin, Berlin, Germany; [5]Department of Paediatrics, Usmanu Danfodiyo University Teaching Hospital, Sokoto, Nigeria.

*saskia.vd.kam@amsterdam.msf.org

The table below indicates the CONSORT recommendations in italics and directly below the section in the manuscript where this can be found. (http://www.consort-statement.org/consort-2010)

Item No		Recommendation
**Title and abstract**	1	(*a*) *Identification as a randomised trial in the title*<br>**Title: "… A Randomised Controlled Trial in Nigeria"**<br>**Abstract: "Methods and Findings"**
		(*b*) Structured summary of trial design, methods, results, and conclusions<br>**Abstract: "Methods and Findings " and "Conclusion"**
**Introduction**		
Background /objectives	2	(*a*) *Scientific background and explanation of rationale*<br>**Introduction section**
		(*b*) *Specific objectives or hypotheses*<br>**"Methods", subsection "*Study Objectives and Endpoints*", paragraph 1**
**Methods**		
Trial design	3	*(a) Description of trial design (such as parallel, factorial) including allocation ratio*<br>**"Methods", subsection "Study Population and Randomisation", Paragraph 2 and 5**
		(*b*) *Important changes to methods after trial commencement (such as eligibility criteria), with reasons*<br>**None**
Participants	4	(*a*) *Eligibility criteria for participants*<br>**"Methods", subsection "Study Population and Randomisation", paragraph 1**
		(*b*) *Settings and locations where the data were collected*

		**"Methods", subsection "Setting", paragraph 1-6**
Interventions	5	*The interventions for each group with sufficient details to allow replication, including how and when they were actually administered*<br>**"Methods", sub section "Procedures" paragraph 1-9**
Outcomes	6	(*a*) *Completely defined pre-specified primary and secondary outcome measures, including how and when they were assessed*<br>**"Methods", sub section "Procedures" paragraph 3-7 and subsection " Study Objectives and Endpoints" paragraph 1**
		(b) Any changes to trial outcomes after the trial commenced, with reasons<br>**none**
Sample size	7	(*a*) *How sample size was determined*<br>**"Methods", sub section "Study Population and Randomisation", paragraph 4**
		(b) When applicable, explanation of any interim analyses and stopping guidelines<br>**N/A**
Randomisation:	8	*(a) Method used to generate the random allocation sequence*<br>**"Methods", sub section "Study Population and Randomisation", paragraph 5**
		(*b*) *Type of randomisation; details of any restriction (such as blocking and block size)*<br>**" Methods", sub section "Study Population and Randomisation", Paragraph 2 and 5**
Allocation	9	*Mechanism used to implement the random allocation sequence (such as sequentially numbered containers), describing any steps taken to conceal the sequence until interventions were assigned*<br>**"Methods", sub section "Study Population and Randomisation", Paragraph 5**
	10	*Who generated the random allocation sequence, who enrolled participants, and who assigned participants to interventions*<br>**"Methods", sub section "Study Population and Randomisation", Paragraph 5**
	11	(*a*) *If done, who was blinded after assignment to interventions (for example, participants, care providers, those assessing outcomes) and how*<br>**"Methods", sub section "Study Population and Randomisation", Paragraph 5**
		(b) If relevant, description of the similarity of interventions<br>**Not relevant**
Statistical methods	12	*(a) Statistical methods used to compare groups for primary and secondary outcomes*<br>**"Methods", sub section "Data analysis", Paragraph 1-2**
		(b) Methods for additional analyses, such as subgroup analyses and adjusted analyses<br>**"Methods" section, sub section "Data analysis", Paragraph 2**
**Results**		

Participants	13	*(a) For each group, the numbers of participants who were randomly assigned, received intended treatment, and were analysed for the primary outcome*<br>**"Result", sub section "Participant flow", Paragraph 1 and Fig.1 Flow chart**
		(b) For each group, losses and exclusions after randomisation, together with reasons<br>**" Result", sub section "Participant flow", Paragraph 1 and Fig.1 Flow chart**.
Recruitment	14	*(a) Dates defining the periods of recruitment and follow-up*<br>**"Result", sub section "Participant flow", Paragraph 1**
		(b) Why the trial ended or was stopped<br>**N/A;**
Baseline data	15	*A table showing baseline demographic and clinical characteristics for each group*<br>**"Result", sub section "Baseline Characteristics" and " Table 2. Baseline Characteristics"**
Numbers analysed	16	*For each group, number of participants (denominator) included in each analysis and whether the analysis was by original assigned groups*<br>**"Result", sub section "Participant flow", Paragraph 1 and "Fig.1 Flow chart" and " Result", sub section "Incidence of Malnutrition", Table 3. Incidence of first NNO (negative nutritional outcome) within one year"**
Outcomes and estimation	17	*(a) For each primary and secondary outcome, results for each group, and the estimated effect size and its precision (such as 95% confidence interval)*<br>**"Result", sub section "Incidence of Malnutrition", Table 3. Incidence of first NNO (negative nutritional outcome) within one year" and Table 4. Incidence of first moderate and severe malnutrition event among non-malnourished at enrolment per year**
		(b) For binary outcomes, presentation of both absolute and relative effect sizes is recommended<br>**As above**
Ancillary analyses	18	*Results of any other analyses performed, including subgroup analyses and adjusted analyses, distinguishing pre-specified from exploratory*<br>**"Result", sub section "Incidence of Malnutrition", Table 5 Incidence rate of first NNO (negative nutritional outcome) per year by subgroups, adjusted for nutritional status at baseline**<br>**Table 1: Anthropometric indicators: change from baseline to day 14 and to day 168**
Harms	19	*All important harms or unintended effects in each group (for specific guidance see CONSORT for harms)*<br>**"Result", sub section "Disease and mortality" and Table 8. Mortality and hospital admissions by intervention group and" Discussion" section, sub section "Risks" paragraph 1-4**
**Discussion**		
Limitations	20	*Trial limitations, addressing sources of potential bias, imprecision, and, if relevant, multiplicity of analyses*

		**“Discussion”, sub section “Limitations”, paragraph 1-5**
Generalisability	21	*Generalisability (external validity, applicability) of the trial findings*<br>**“Discussion”, sub section “Limitations”, paragraph 5**
Interpretation	22	*Interpretation consistent with results, balancing benefits and harms, and considering other relevant evidence*<br>**“Discussion”, sub section “Incidence of malnutrition”, paragraph 1-*5*.**”
**Other information**		
Registration	23	*Registration number and name of trial registry*<br>**“Methods”, sub section “Approval”, paragraph 1**
Protocol	24	*Where the full trial protocol can be accessed, if available*<br>**“Methods”, sub section “Approval”, paragraph 1**
Funding	25	*Sources of funding and other support (such as supply of drugs), role of funders*<br>**Funding sources are mentioned on website of PlosMed**.
